# Abstracts from the 4th ImmunoTherapy of Cancer Conference: Prague, Czech Republic. 20–22 March 2017

**DOI:** 10.1186/s40425-017-0219-4

**Published:** 2017-03-20

**Authors:** J. Ženka, V. Caisová, O. Uher, P. Nedbalová, K. Kvardová, K. Masáková, G. Krejčová, L. Paďouková, I. Jochmanová, K. I. Wolf, J. Chmelař, J. Kopecký, L. Loumagne, J. Mestadier, S. D’agostino, A. Rohaut, Y. Ruffin, V. Croize, O. Lemaître, S. S. Sidhu, S. Althammer, K. Steele, M. Rebelatto, T. Tan, T. Wiestler, A. Spitzmueller, R. Korn, G. Schmidt, B. Higgs, X. Li, L. Shi, X. Jin, K. Ranade, S. Koeck, A. Amann, G. Gamerith, M. Zwierzina, E. Lorenz, H. Zwierzina, J. Kern, M. Riva, T. Baert, A. Coosemans, R. Giovannoni, E. Radaelli, W. Gsell, U. Himmelreich, M. Van Ranst, F. Xing, W. Qian, C. Dong, X. Xu, S. Guo, Q. Shi, D. Quandt, B. Seliger, C. Plett, D. C. Amberger, A. Rabe, D. Deen, Z. Stankova, A. Hirn, Y. Vokac, J. Werner, D. Krämer, A. Rank, C. Schmid, H. Schmetzer, M. Guerin, J. M. Weiss, F. Regnier, G. Renault, L. Vimeux, E. Peranzoni, V. Feuillet, M. Thoreau, T. Guilbert, A. Trautmann, N. Bercovici, D. C. Amberger, F. Doraneh-Gard, C. L. Boeck, C. Plett, C. Gunsilius, C. Kugler, J. Werner, J. Schmohl, D. Kraemer, B. Ismann, A. Rank, C. Schmid, H. M. Schmetzer, A. Markota, C. Ochs, P. May, A. Gottschlich, J. Suárez Gosálvez, C. Karches, D. Wenk, S. Endres, S. Kobold, T. Hilmenyuk, R. Klar, F. Jaschinski, G. Gamerith, F. Augustin, E. Lorenz, C. Manzl, E. Hoflehner, P. Moser, B. Zelger, S. Köck, A. Amann, J. Kern, G. Schäfer, D. Öfner, H. Maier, H. Zwierzina, S. Sopper, H. Prado-Garcia, S. Romero-Garcia, R. Sandoval-Martínez, A. Puerto-Aquino, J. Lopez-Gonzalez, U. Rumbo-Nava, R. Klar, T. Hilmenyuk, F. Jaschinski, A. Coosemans, T. Baert, A. Van Hoylandt, P. Busschaert, I. Vergote, T. Baert, A. Van Hoylandt, P. Busschaert, I. Vergote, A. Coosemans, J. Laengle, K. Pilatova, E. Budinska, B. Bencsikova, R. Sefr, R. Nenutil, V. Brychtova, L. Fedorova, B. Hanakova, L. Zdrazilova-Dubska, Chris Allen, Yuan-Chieh Ku, Warren Tom, Yongming Sun, Alex Pankov, Tim Looney, Fiona Hyland, Janice Au-Young, Ann Mongan, A. Becker, J. B. L. Tan, A. Chen, K. Lawson, E. Lindsey, J. P. Powers, M. Walters, U. Schindler, S. Young, J. C. Jaen, S. Yin, Y. Chen, I. Gullo, G. Gonçalves, M. L. Pinto, M. Athelogou, G. Almeida, R. Huss, C. Oliveira, F. Carneiro, C. Merz, J. Sykora, K. Hermann, R. Hussong, D. M. Richards, H. Fricke, O. Hill, C. Gieffers, M. P. Pinho, J. A. M. Barbuto, S. E. McArdle, G. Foulds, J. N. Vadakekolathu, T. M. A. Abdel-Fatah, C. Johnson, S. Hood, P. Moseley, R. C. Rees, S. Y. T. Chan, A. G. Pockley, S. Rutella, C. Geppert, A. Hartmann, K. Senthil Kumar, M. Gokilavani, S. Wang, C. Merz, D. M. Richards, J. Sykora, M. Redondo-Müller, K. Heinonen, V. Marschall, M. Thiemann, H. Fricke, C. Gieffers, O. Hill, L. Zhang, B. Mao, Y. Jin, G. Zhai, Z. Li, Z. Wang, W. Qian, X. An, M. Qiao, J. Zhang, Q. Shi, J. Weber, H. Kluger, R. Halaban, M. Sznol, H. Roder, J. Roder, J. Grigorieva, S. Asmellash, C. Oliveira, K. Meyer, A. Steingrimsson, S. Blackmon, R. Sullivan, C. L. Boeck, D. C. Amberger, F. Doraneh-Gard, W. Sutanto, T. Guenther, J. Schmohl, F. Schuster, H. Salih, F. Babor, A. Borkhardt, H. Schmetzer, Y. Kim, I. Oh, C. Park, S. Ahn, K. Na, S. Song, Y. Choi, L. Fedorova, A. Poprach, R. Lakomy, I. Selingerova, R. Demlova, K. Pilatova, S. Kozakova, D. Valik, K. Petrakova, R. Vyzula, L. Zdrazilova-Dubska, D. Aguilar-Cazares, M. Galicia-Velasco, C. Camacho-Mendoza, L. Islas-Vazquez, R. Chavez-Dominguez, C. Gonzalez-Gonzalez, H. Prado-Garcia, J. S. Lopez-Gonzalez, S. Yang, K. D. Moynihan, M. Noh, A. Bekdemir, F. Stellacci, D. J. Irvine, B. Volz, K. Kapp, D. Oswald, B. Wittig, M. Schmidt, R. Chavez-Dominguez, D. Aguilar-Cazares, H. Prado-Garcia, L. Islas-Vazquez, J. S. Lopez-Gonzalez, R. Kleef, A. Bohdjalian, D. McKee, R. W. Moss, Mesha Saeed, Sara Zalba, Reno Debets, Timo L. M. ten Hagen, S. Javed, J. Becher, F. Koch-Nolte, F. Haag, E. M. Gordon, K. K. Sankhala, N. Stumpf, W. Tseng, S. P. Chawla, N. González Suárez, G. Bergado Báez, M. Cruz Rodríguez, A. Gutierrez Pérez, L. Chao García, D. Hernández Fernández, J. Raymond Pous, B. Sánchez Ramírez, C. Jacoberger-Foissac, H. Saliba, C. Seguin, A. Brion, B. Frisch, S. Fournel, B. Heurtault, T. Otterhaug, M. Håkerud, A. Nedberg, V. Edwards, P. Selbo, A. Høgset, T. Jaitly, J. Dörrie, N. Schaft, S. Gross, B. Schuler-Thurner, S. Gupta, L. Taher, G. Schuler, J. Vera, F. Rataj, F. Kraus, S. Grassmann, M. Chaloupka, S. Lesch, C. Heise, S. Endres, S. Kobold, B. M. Loureiro Cadilha, K. Dorman, C. Heise, F. Rataj, S. Endres, S. Kobold

**Affiliations:** 1 Department of Medical Biology, Faculty of Science, University of South Bohemia, Česke Budějovice, Czech Republic; 2 1st Department of Internal Medicine, Medical Faculty of P. J. Šafárik University in Košice, Košice, Slovakia; 3 University of Michigan Medical Center, Ann Arbor, MI USA; 4 Sanofi, Vitry, France; 5 DEFINIENS, Muenchen, Germany; 6 Medimmune, Gaithersburg, MD USA; 7 Medical University of Innsbruck, Department of Internal Medicine V, Innsbruck, Austria; 8 Medical University of Innsbruck, Department of Plastic, Reconstructive and Aesthetic Surgery, Innsbruck, Austria; 9 Tyrolean Cancer Research Institute, Innsbruck, Austria; 10 Department of Oncology, Laboratory of Tumor Immunology and Immunotherapy, KU Leuven, Leuven, Belgium; 11 Gynaecology and Obstetrics, Leuven Cancer Institute, UZ Leuven, Leuven, Belgium; 12 Department of Surgical Sciences, University of Milano Bicocca, Monza, Italy; 13 Center for the Biology of Disease, KU Leuven Center for Human Genetics - InfraMouse, VIB, University of Leuven, Leuven, Belgium; 14 Biomedical MRI, Department of Imaging and Pathology, KU Leuven, Leuven, Belgium; 15 Laboratory for Clinical and Epidemiological Virology, Rega Institute for Medical Research, KU Leuven, Leuven, Belgium; 16 Crown Biosciences, Taicang, Jiangsu China; 17 University of Halle, Halle, Germany; 18 University Hospital of Munich, Department of Hematopoetic Cell Transplantation, Med.Dept. III, 81377 Munich, Germany; 19 University Hospital of Tuebingen, Department of Hematology and Oncology, 72076 Tuebingen, Germany; 20 Municipal Hospital Oldenburg, Department of Hematology and Oncology, 26133 Oldenburg, Germany; 21 Municipal Hospital Augsburg, Dept of Medicine II, Section of Hematopoetic Cell Transplantations, 86156 Augsburg, Germany; 22 Cochin Institute, Paris, France; 23 Inserm U1016 CNRS UMR 8104 Univ Paris Descartes, Paris, France; 24 Univ Medical Center Freiburg, Freiburg, Germany; 25 University Hospital of LMU, Department for Hematopoietic cell transplantation, Med3, Munich, Germany; 26 University Hospital of Tuebingen, Department for Hematology and Oncology, Tuebingen, Germany; 27 University Hospital of Oldenburg, Department for Hematology and Oncology, Oldenburg, Germany; 28 University Hospital of LMU, Department for Hematology and Oncology, Med3, Munich, Germany; 29 Municipal Hospital of Augsburg, Department of Hematology and Oncology, Augsburg, Germany; 30 Divison of Clinical Pharmacology, Department of Internal Medicine IV, Ludwig-Maximilians Universität, Munich, Germany; 31 Secarna Pharmaceuticals GmbH & Co. KG, Planegg/Martinsried, Germany; 32 Clinic of Internal Medicine V, Medical University of Innsbruck, Innsbruck, Austria; 33 Department of Visceral, Transplant and Thoracic Surgery, Medical University of Innsbruck, Innsbruck, Austria; 34 Tyrolean Cancer Research Institute, Innsbruck, Austria; 35 Department of Pathology, Medical University of Innsbruck, Innsbruck, Austria; 36 National Institute of Respiratory Diseases, Mexico City, Mexico; 37 Secarna pharmaceuticals GmbH & Co KG, Martinsried, Germany; 38 KU Leuven, Department of Oncology, Laboratory of Tumor Immunology and Immunotherapy, Immunovar Research Group, Leuven, Belgium; 39 UZ Leuven, Gynaecology and Obstetrics, Leuven Cancer Institute, Leuven, Belgium; 40 KU Leuven, Department of Oncology, Laboratory of Gynaecologic Oncology, Leuven, Belgium; 41 KU Leuven, Department of Oncology, Laboratory of Tumor Immunology and Immunotherapy, Immunovar Research Group, Leuven, Belgium; 42 UZ Leuven, Gynaecology and Obstetrics, Leuven Cancer Institute, Leuven, Belgium; 43 KU Leuven, Department of Oncology, Laboratory of Gynaecologic Oncology, Leuven, Belgium; 44 Department of Surgery, Vienna, Austria; 45 Masaryk Memorial Cancer Institute, Brno, Czech Republic; 46 Faculty of Medicine Masaryk University, Brno, Czech Republic; 47 Faculty of Science Masaryk University, Brno, Czech Republic; 48 Thermofisher Scientific, South San Francisco, CA USA; 49 ARCUS Biosciences, Hayward, CA USA; 50 Medizinische Klinik und Poliklinik IV, Klinikum der Universität München, Muenchen, Germany; 51 Institute of Molecular Pathology and Immunology at the University of Porto (Ipatimup) and Institute for Research Innovation in Health (i3S), Porto, Portugal; 52 Department of Pathology and Oncology, Faculty of Medicine of the University of Porto, Porto, Portugal; 53 Centro Hospitalar de São João, Porto, Portugal; 54 Department of Biomedical Sciences and Medicine, University of Algarve, Algarve, Portugal; 55 INEB-Institute of Biomedical Engineering, University of Porto, Porto, Portugal; 56 ICBAS-Institute of Biomedical Sciences Abel Salazar, University of Porto, Porto, Portugal; 57 Definiens AG, Munich, Germany; 58 Department of Pathology and Oncology, Faculty of Medicine of the University of Porto, Porto, Portugal; 59 Apogenix AG, 69120 Heidelberg, Germany; 60 Biomedical Sciences Institute, University of Sao Paulo, Sao Paulo, Brazil; 61 Cell and Molecular Therapy Center, NUCEL-NETCEM, University of Sao Paulo, Sao Paulo, Brazil; 62 Nottingham Trent University, John van Geest Cancer Research Centre, Nottingham, United Kingdom; 63 Clinical Oncology Department, Nottingham University Hospital NHS Trust, Nottingham, United Kingdom; 64 Institute of Pathology, Erlangen, Germany; 65 National Chung Hsing University, Taichung, Taiwan; 66 Academia Sinica, Taipei, Taiwan; 67 Apogenix AG, 69120 Heidelberg, Germany; 68 Crown Bioscience Inc., Taicang, China; 69 NYU Langone Medical Center, New York, NY USA; 70 Yale University School of Medicine, New Haven, CT USA; 71 Biodesix, Inc., Boulder, CO USA; 72 Massachusetts General Hospital, Boston, MA USA; 73 University Hospital of Munich, Department of Hematopoetic Cell Transplantation, Med. Dept. 3, Munich, Germany; 74 University Hospital of Tuebingen, Department of Hematology and Oncology, Tuebingen, Germany; 75 University Hospital of Duesseldorf, Department of Pediatric Hematology, Oncology and Clinical Immunology, Duesseldorf, Germany; 76 Chonnam National University Hwasun Hospital, Jeonnam, Republic of Korea; 77 Masaryk Memorial Cancer Institute, Brno, Czech Republic; 78 Faculty of Medicine Masaryk University, Brno, Czech Republic; 79 Faculty of Science Masaryk University, Brno, Czech Republic; 80 Instituto Nacional de Enfermedades Respiratorias Ismael Cosio Villegas, Mexico City, Mexico; 81 Facultad de Ciencias, Universidad Nacional Autonoma de Mexico, Mexico City, Mexico; 82 Koch Institute, MIT, Cambridge, MA USA; 83 Biological Engineering, MIT, Cambridge, MA USA; 84 Institute of Materials, EPFL, Lausanne, Switzerland; 85 Mologen AG, Berlin, Germany; 86 Foundation Institute Molecular Biology and Bioinformatics, Freie Universitaet Berlin, Berlin, Germany; 87 Instituto Nacional de Enfermedades Respiratorias Ismael Cosio Villegas, Mexico City, Mexico; 88 Integrative Oncology, Vienna, Austria; 89 Rudolfinerhaus, Vienna, Austria; 90 San Diego Cancer Research Institute, San Diego, CA USA; 91 Cancer Decisions, Lemont, PA USA; 92 Laboratory of Experimental Surgical Oncology, Section Surgical Oncology, Department of Surgery, Erasmus MC, Rotterdam, The Netherlands; 93 Laboratory of Tumor Immunology, Department of Medical Oncology, Erasmus MC Cancer Institute, Rotterdam, The Netherlands; 94 Institute of Immunology, University Medical Center Hamburg-Eppendorf, Hamburg, Germany; 95 Sarcoma Oncology Center/Cancer Center of Southern California, Santa Monica, CA USA; 96 University of Southern California Keck School of Medicine, Los Angeles, CA USA; 97 Center of Molecular Immunology, Havana, Cuba; 98 Equipe de Biovectorologie, Laboratoire de Conception et Application de Molécules Bioactives, Faculté de Pharmacie, Illkirch, France; 99 PCI Biotech, Oslo, Norway; 100 Oslo University Hospital, Oslo, Norway; 101 Fredrich Alexander University Hospital, Erlangen, Germany; 102 University of Rostock, Rostock, Germany; 103 Fredrich Alexander University, Erlangen, Germany; 104 Center of Integrated Protein Science Munich (CIPS-M) and Division of Clinical Pharmacology, Department of Internal Medicine IV, Klinikum der Universität München, Munich, Germany; 105 Member of the German Center for Lung Research, Munich, Germany; 106 Division of Clinical Pharmacology, Department of Medicine IV, Klinikum der Universität München, Munich, Germany

## Preclinical models

### A1 Intratumoral immunotherapy. B16-F10 murine melanoma model

#### J. Ženka^1^, V. Caisová^1^, O. Uher^1^, P. Nedbalová^1^, K. Kvardová^1^, K. Masáková^1^, G. Krejčová^1^, L. Paďouková^1^, I. Jochmanová^2^, K. I. Wolf^3^, J. Chmelař^1^, J. Kopecký^1^

##### ^1^Department of Medical Biology, Faculty of Science, University of South Bohemia, Česke Budějovice, Czech Republic; ^2^1st Department of Internal Medicine, Medical Faculty of P. J. Šafárik University in Košice, Košice, Slovakia; ^3^University of Michigan Medical Center, Ann Arbor, MI, United States

###### **Correspondence:** J. Ženka

**Background:** Cancer immunotherapy based on direct intratumoral injection of immunomodulators has been established at the end of 19th century using Coley’s toxin. Our novel therapeutic strategy is based on the intratumoral injection of optimized mixture of TLR agonists, causing strong inflammatory infiltration. Infiltrating cells (mainly phagocytes) are directed to artificially opsonized tumor cells covered by phagocytosis stimulating ligands.

**Materials and methods:** Immunotherapy was tested using B16-F10 murine melanoma model. Inflammatory infiltration was achieved using the mixture of resiquimod, poly(I:C), and lipoteichoic acid. Artificial opsonisation of tumor cells was elicited by mannan anchored to cell membranes using a hydrophobic anchor. The course of tumor infiltration was studied using flow cytometry. Cytotoxic effect of infiltrating immune cells on opsonized tumor cells was studied *in vitro*. Participation of acquired immunity was elucidated on the basis of intracellular IFN-gamma production by lymphocytes.

**Results:** Optimized cancer immunotherapy based on the synergy of TLR agonists with artificially induced tumor opsonisation resulted in complete cure of 83% of mice with advanced melanomas. Moreover, cured mice acquired resistance to retransplantation of tumor cells. Applied therapy has a strong antimetastatic effect. In the first phase of immunotherapy, granulocyte predominance was observed, followed by involvement of acquired immunity.

**Conclusions:** Intratumoral immunotherapy initiated by innate immunity based attack followed by joining of mechanisms of acquired immunity is a promising approach for future application in clinical practice as compounds used are safe for humans.

### A2 Assessing humanized mouse models for cancer immunotherapy

#### L. Loumagne, J. Mestadier, S. D’agostino, A. Rohaut, Y. Ruffin, V. Croize, O. Lemaître, S. S. Sidhu

##### Sanofi, Vitry, France

###### **Correspondence:** L. Loumagne

**Background:** Preclinical models that can recapitulate a functional human immune system are essential tools for the continued investigation of novel immunotherapy approaches. In this regard, immunodeficient mice offer a unique opportunity to reconstitute, at least partially, a human immune system together with human tumors to characterize immunomodulator compounds in a more physiologically relevant setting. The aim of this project was to perform a head to head comparison of several highly immunodeficient mouse strains engrafted with human CD34+ Hematopoietic Stem Cells (HSCs) for their ability to develop and sustain a multi-lineage engraftment of human immune cells. The most promising mouse model(s) will then be assessed for their capacity to support human tumor xenografts (CDX and PDX). Finally, we will determine if we can elicit an immune response to selected immunotherapeutic agents to drive anti-tumor activity.

**Materials and methods:** The mice strains analyzed are NSG, NOG, NSG-SGM3, and NOG-EXL; the latter two being transgenic for the expression of human cytokines important in myeloid cell development. Humanization was performed in-house by injecting human umbilical cord blood CD34+ HSCs from different donors into the tail-vein of 4–5 week old female mice post-irradiation. To monitor human immune system reconstitution, flow cytometry analysis on blood was performed at approximately 4 week intervals beginning at week 4 post-injection of human CD34+ HSCs. At week 18, a terminal analysis was performed, consisting of a thorough immune cell profiling of blood, spleen and bone marrow by flow cytometry. Tissues were collected for the immunohistochemical staining of human immune cells and ex vivo functional assays will be performed.

**Results:** Comparison of the immune system from different humanized mouse models show that all strains display high levels of engraftment of human CD45+ cells ranging from 50 to 70% at 18 weeks post-humanization. Population such as T CD4+ and CD8+ as well as B cells are well reconstituted and minor populations such as monocytes/macrophages, NK, and subsets of DC are also present at a lower percentage. Complete results obtained from different mouse strains will be available on the congress day.

**Conclusions:** Overall, this project will provide a head-to-head comparison of several humanized mouse models that will be of value in the immunotherapy field according to the potent advantages of these models and the paucity of studies directly comparing different immunodeficient mouse strains.

### A3 A predictive PD-L1*CD8 cell density score for NSCLC patients treated with durvalumab

#### S. Althammer^1^, K. Steele^2^, M. Rebelatto^2^, T. Tan^1^, T. Wiestler^1^, A. Spitzmueller^1^, R. Korn^1^, G. Schmidt^1^, B. Higgs^2^, X. Li^2^, L. Shi^2^, X. Jin^2^, K. Ranade^2^

##### ^1^DEFINIENS, Muenchen, Germany; ^2^Medimmune, Gaithersburg, MD, United States

###### **Correspondence:** S. Althammer

**Background:** Current PD-1/PD-L1 checkpoint inhibitor based immuno-therapies for advanced non-small cell lung cancer (NSCLC) show impressive response rates and long survival. We used automated image^1^ and data analysis of immunohistochemically (IHC) stained tissue sections within the Tissue Phenomics methodology to determine whether CD8+ and PD-L1+ cells densities could better identify patients most likely to respond to durvalumab than using a manual PD-L1 scoring of positive tumor cells (25% cutoff). Durvalumab is a human IgG1 monoclonal antibody that inhibits programmed death ligand-1 (PD-L1) binding to programmed death-1 and B7.1/CD80, restoring antitumor immunity^2,3^.

**Materials and methods:** CP1108/NCT01693562 was a nonrandomized phase 1/2 trial evaluating durvalumab in advanced NSCLC and other solid tumors^4^. Digital slides from 163 baseline tumor biopsies, IHC stained for PD-L1 (Ventana SP263) and CD8 (Ventana SP239), were fully automatically scored with Definiens’ Developer XD 2.1.4 software using the product of PD-L1+ and CD8+ cell densities. The image analysis results were quality controlled using ground truth annotations provided by a panel of three pathologists. A discovery set (n = 84) was used to identify the scoring algorithm and the cutoff value, which was then tested on a validation set (n = 79). The robustness of the scoring algorithm was pre-validated using Monte-Carlo (MC) cross-validation.

**Results:** The most accurate and robust scoring method in terms of MC performance metrics (positive predictive value, prevalence, pOS, pPFS) was identified as the product of average CD8+ and PD-L1+ cells densities in the pathologist annotated tumor center. Patients with high CD8*PD-L1 scores show significantly improved outcome compared to the CD8*PD-L1 low subgroup (ORR: 0.39 vs 0.07, OS log rank p-value: 0.0099 (training set) and 0.00053 (confirmation set)). CD8*PD-L1 further appears to predict patients’ outcome better than the PD-L1 status visually determined by pathologists (ORR: 39% vs 27%, median OS: 24.3 vs 17.8).

**Conclusions:** Although further validation studies are required, we observe that a scoring algorithm based on automated image analysis of CD8+ and PDL1+ cell densities in baseline tumor biopsies improves the stratification of NSCLC patients treated with durvalumab when compared to visual histopathology scoring.

**References**

1. Brieu, et al. Proc. SPIE 9784 Medical Imaging 2016: doi:10.1117/12.2208620.

2. MedImmune/AstraZeneca. Data on file.

3. Ibrahim R, et al. Semin Oncol 2015;42(3):474–83.

4. Rizvi NA, et al. J Clin Oncol 2015;33(Suppl.):[Abstract 8032].

### A4 The influence of specific cytokines on cancer microtissue infiltrating cytotoxic T lymphocyte subpopulations

#### S. Koeck^1^, A. Amann^1^, G. Gamerith^1^, M. Zwierzina^2^, E. Lorenz^3^, H. Zwierzina^1^, J. Kern^3^

##### ^1^Medical University of Innsbruck, Department of Internal Medicine V, Innsbruck, Austria; ^2^Medical University of Innsbruck, Department of Plastic, Reconstructive and Aesthetic Surgery, Innsbruck, Austria; ^3^Tyrolean Cancer Research Institute, Innsbruck, Austria

###### **Correspondence:** S. Koeck

**Background:** Cancer associated fibroblasts are known to highly interact with infiltrating immune cells within the tumor microenvironment. In this work, we investigate the influence of specific fibroblast derived cytokines on the infiltration capacity of CD3 + CD8+ cytotoxic T lymphocyte subpopulations using a multicellular 3D co-culture System.

**Materials and methods:** 3D tumor microtissues were cultivated using a hanging drops system. Human A549 and Calu-6 cancer cell lines were incubated alone or together with the human fibroblast cell line SV80 for 10 days to form microtissues. On day 10, peripheral blood mononuclear cells (PBMC) were added for 24 h. Endogenous cytokine expression of interleukin-2 (IL-2), IL-4, IL-5, IL-6, IL-12p70, interferon gamma (IFN-γ) and tumor necrosis factor alpha (TNF-α) in microtissue mono-, co- and tri-cultures was analyzed with a multi-cytokine immunoassay. Subsequently, measured cytokines were added to cancer cell/PBMC co-cultures to investigate the effect of these cytokines on infiltrating immune cell subpopulations. Infiltrating subpopulations were investigated by flow cytometry.

**Results:** In both A549/SV80 and Calu-6/SV80 co-cultures and in SV80 monocultures, the cytokines TNF-α, IL-2, IL-5, IL-6 and IL-12p70 were measured. Minimal or no concentrations of the investigated cytokines were detected in cancer cell and PBMC monocultures. IL-4 and IFN-γ were not measured in all approaches. Additional application of the detected cytokines showed that IL-2 inhibited infiltration of naïve T lymphocytes into cancer cell/PBMC co-cultures and enhanced infiltration of differentiated T lymphocytes. Application of IL-5, IL-6 and TNF-α had only minor effects on infiltrating T lymphocyte subpopulations.

**Conclusions:** We show that in multicellular cancer microtissues, cancer associated fibroblasts are a main origin of cytokines. Moreover, we prove that IL-2 increases the infiltration of differentiated T lymphocytes. Targeting the interaction of T lymphocyte subpopulations and cytokines derived from cancer associated fibroblasts might be a promising strategy for future therapeutic approaches.

### A5 A strategy to improve the translational impact of murine high grade glioma

#### M. Riva^1^, T. Baert^1,2^, A. Coosemans^1,2^, R. Giovannoni^3^, E. Radaelli^4^, W. Gsell^5^, U. Himmelreich^5^, M. Van Ranst^6^

##### ^1^Department of Oncology, Laboratory of Tumor Immunology and Immunotherapy, KU Leuven, Leuven, Belgium; ^2^Gynaecology and Obstetrics, Leuven Cancer Institute, UZ Leuven, Leuven, Belgium; ^3^Department of Surgical Sciences, University of Milano Bicocca, Monza, Italy; ^4^Center for the Biology of Disease, KU Leuven Center for Human Genetics - InfraMouse, VIB, University of Leuven, Leuven, Belgium; ^5^Biomedical MRI, Department of Imaging and Pathology, KU Leuven, Leuven, Belgium; ^6^Laboratory for Clinical and Epidemiological Virology, Rega Institute for Medical Research, KU Leuven, Leuven, Belgium

###### **Correspondence:** M. Riva

**Background:** High-grade gliomas (HGGs) are one of the deadliest cancers. In the last decade, immunotherapies have shown promising results in preclinical studies; however, when translated into clinical practice, they did not provide the expected outcomes. The low translational impact of animal models used in such preclinical studies is one of the main causes of discrepancy. These models often lack a well characterized glioma stem-cells (GSCs) compartment, which is one of the major causes of immune escape and resistance to standard therapy. Moreover, the immune status of the animals has never been characterized nor considered as a significant factor, despite the well-known immunosuppression in HGG patients. The aim of this study was to develop a new preclinical model of HGG, based on the recent in vitro demonstration that neurosphere (NS) culture is effective in increasing the amount of GSCs in the CT-2A cell-line of murine HGG. In this model, a special focus was given to GSCs and to the immune status of the mice in order to overcome the above mentioned limitations.

**Materials and methods:** CT-2A NS were established from standard monolayer (ML) cultures. Orthotopic tumors were generated by inoculating the brain of C57BL/6 mice with 5000 CT-2A cells, derived either from ML or NS cultures. The expression of stemness markers (Oct3/4, Nestin and CD133) was evaluated with flow-cytometry and immunohistochemistry in cell cultures and in brain tumors. Tumor volume was assessed with 9.4 T MRI at serial time points. The immune status of the mice was evaluated with cytometric bead array of circulating cytokines known for promoting tumor growth (TNFalpha, G-CSF, IL-1B and CCL-2), or mediating the anti-tumor response (IL-2 and IFNgamma).

**Results:** Flow-cytometry and immunohistochemistry showed a higher expression of stemness markers in NS cultures compared to ML. This difference persisted after in vivo inoculation of tumor cells: compared to ML, NS tumors showed a higher expression of Oct3/4 (p < 0,01), Nestin (p < 0,001) and CD133 (p < 0,05). NS tumors were more aggressive than their ML counterpart, as they displayed higher mitotic index (p < 0,0001) and increased vascular proliferation (p < 0,001). As a consequence, mice inoculated with NS cells survived significantly shorter (p < 0,005) and developed larger tumors (p < 0,01). In preliminary experiments, changes were also found for circulating cytokines. TNFalfa, G-CSF, IL-1B and CCL-2, which are increased in HGG patients, displayed higher levels in NS tumors compared to ML (42,6 vs 27,0 pg/mL; 63,1 vs 34,9 pg/mL; 39,0 vs 24,1 pg/mL; 111,8 vs 84,7 pg/mL). Similarly, the level of the anti-tumor immune response cytokines in NS tumors was in line with what is observed in the clinical setting: IL-2 increased (7,5 vs 4,1 pg/mL), IFNgamma decreased (1,0 vs 2,0 pg/mL) in comparison with ML tumors.

**Conclusions:** This work represents the first demonstration that brain tumors generated from CT-2A NS retain an enriched population of GSCs; furthermore, these tumors induce in the host mice immunological alterations that closely mimic the clinical situation. This new experimental platform offers a unique opportunity to study new immunotherapeutic approaches for HGGs, allowing to test their therapeutic effect on the GSC compartment in the context of the specific immunological alterations which characterize HGG patients.

### A6 Genomic profiling and in vitro screen of syngeneic mouse cell lines testing checkpoint inhibitors in combination with targeted agents in a coculture system with mouse T cells

#### F. Xing, W. Qian, C. Dong, X. Xu, S. Guo, Q. Shi

##### Crown Biosciences, Taicang, Jiangsu, China

###### **Correspondence:** F. Xing

**Background:** Cancer immunotherapy has provided substantial clinical benefits to patients with advanced diseases. In pre-clinical studies, CrownBio has developed a large collection of syngeneic models to facilitate *in vivo* efficacy testing of immune-oncology agents in mice (MuScreen^TM^). To combine the ostensibly separate therapeutic strategies of activating immune cells against and targeting the unique genetic characteristics of a tumor model, we sought to thoroughly characterize the mutation profiles of these syngeneic mouse cell lines and examine drug response profiles of these cell line models. The goal of this work was to provide an *in vitro* system in evaluating combination effectiveness when targeting both immune checkpoint markers and oncogenic targets in preclinical studies.

**Materials and methods:** We investigated mutation and gene expression profiles of 18 mouse cancer cell lines out of the 23 syngeneic mouse models for 50 well defined cancer-related genes by RNAseq (Illumina HiSeq X10). Next, we performed in vitro screen of the 18 syngeneic mouse cancer cell lines against aPD1 and aPDL1 antibodies and a few targeted agents as single-agent to generate baseline data of cell growth inhibition (IC50). Finally, we performed a combination assay on the same panel of the 18 syngeneic mouse cell models to examine synergistic effect of PD-1 and PDL1 blockade with targeted small molecules in a co-culture system in the presence of mouse T cells. An IncuCyte real-time imaging platform was used to distinguish activities of T cells and tumor cells.

**Results:** The oncogenic mutations we identified among 30,690 variants in exonic regions of the 50 well characterized oncogenes and tumor suppressors include ALK (3 - frequency, same for the rest), BRAF (4), BRCA1 (7), BRCA2 (12), EGFR (3), ERBB2 (6), EGFR3 (2), FBXW7 (10), FLT3 (12), HRAS (1), KRAS (8), NRAS (1), PDGFRA (11), PTCH1 (9), PIK3CA (2), PTEN (6), RET (3), SETD2 (5), SMAD4 (3), SMO (13), TRP53 (13), TSC1 (3), and TSC2 (10). All of these genetic alterations are clinically actionable. The same set of genes were also subject to mRNA expression change analysis. The in vitro screen results of the panel of mouse cell lines against aPD1 and aPDL1 antibodies and chemo and targeted agents either as single agent or in combination, and the implications in preclinical studies, will be presented and discussed.

**Conclusions:** The future for immune-oncology therapy is in undoubtedly combination therapy. The in vitro screen platform we established here for syngeneic mouse cell lines in a co-culture system with mouse T cells allows quick and cost-efficient screen of checkpoint inhibition agents either alone or with conventional chemo or targeted therapy. Our future plan is to further expand the panel of well annotated syngeneic mouse cell models for the in vitro screen and compare in vitro data with the results of corresponding in vivo studies (MuScreen^TM^).

### A7 Doxorubicin increases TLR4 triggered activation marker on dendritic cells independent of exCalcium and the inflammasome

#### D. Quandt, B. Seliger

##### University of Halle, Halle, Germany

###### **Correspondence:** D. Quandt

**Background:** Low dose chemotherapy alone or in combination with immune checkpoint inhibitors is implemented in clinic routine cancer treatment regimes. Thereby chemotherapy not only has a direct effect on cancer cells but also has proven to indirectly activate the immune system by ICD (immunogenic cell death) of cancer cells and to have direct effects on cells of the innate and adaptive immunity. Furthermore, the success of ICD has been demonstrated to dependent on the ability of DC (dendritic cells) to mount an inflammasome response. ExCalcium has been shown to function as new DAMP (danger associated molecular pattern) activating the inflammasome when combined with TLR4 triggering signals. The ICD triggers immune cell activation by different mediators, among them are ligands for TLR4.

**Materials and methods:** In this study we tested the direct effect of Doxorubicin on activation marker (FACS) and IL-1 beta production (ELISA) has hallmarks of effector responses of BMDC (bone marrow derived dendritic cells) of wildtype, gprc6a ko (calcium receptor) and asc ko (adapter essential for the inflammasome clustering) mice *in vitro*.

**Results:** Previously we identified a lack of response of chemotherapy induced tumor control *in vivo* in gprc6a ko and asc ko mice. Here, the *in vitro* data show a dose dependent robust (CD40 and CD86) and minor (MHC class I and II) increase of expression on dendritic cells when doxorubicin is combined with LPS in wildtype mice. Doxorubicin has no effect on marker expression when added alone. The increase on activation marker by doxorubicin is independent of IL-1 beta production, since no difference on secreted IL-1 beta was seen. Interesting, doxorubicin does not lead to an IL-1 beta production on it’s own or in combination with LPS, as has been suggested earlier. The marked increase in activation marker expression on BMDCs upon Doxorubicin is independent of gprc6a and asc as data revealed no difference. Of note, BMDCs of asc ko mice do not mount an inflammasome response by TLR4 trigger combined with DAMP signals, still the activation marker are highly regulated upon this trigger.

**Conclusions:** Hence, our in *vitro* data reveal an inflammasome and exCalcium signal independent direct activation of dendritic cells by chemotherapy that does not explain the lack of chemo-responsiveness in gprc6a ko mice *in vivo*.

### A8 Immunomodulatory Kits do not induce AML-blasts’ proliferation ex vivo: IPO-38 is an appropriate and reliable marker to detect and quantify proliferating blasts

#### C. Plett^1^, D. C. Amberger^1^, A. Rabe^1^, D. Deen^1^, Z. Stankova^1^, A. Hirn^1^, Y. Vokac^1^, J. Werner^2^, D. Krämer^3^, A. Rank^4^, C. Schmid^4^, H. Schmetzer^1^

##### ^1^University Hospital of Munich, Department of Hematopoetic Cell Transplantation, Med. Dept. III, 81377, Munich, Germany; ^2^University Hospital of Tuebingen, Department of Hematology and Oncology, 72076 Tuebingen, Germany, Tuebingen, Germany; ^3^Municipal Hospital Oldenburg, Department of Hematology and Oncology, 26133 Oldenburg, Oldenburg, Germany; ^4^Municipal Hospital Augsburg, Dept of Medicine II, Section of Hematopoetic Cell Transplantations, 86156 Augsburg, Augsburg, Germany

**Background:** AML-blasts can be converted to DCleu by immunomodulatory ‘Kits’ (ex vivo). T-cells’ anergy can be overcome after stimulation with DC/DCleu and results in antileukemic reactivity. A potential induction of blast-proliferation (e.g. by immunomodulatory Kit-application in vivo) in AML-pts has to be excluded.

**Materials and methods:** 8 ‘Kits’ containing combinations of GM-CSF with 1–2 additional factors (PGE-1/2, Picibanil, IFNɑ, TNFɑ, Calciumionophore) were studied with respect to the generation of DC/DCleu from blasts, mediation of antileukemic reactivity (after DC/DCleu-stimulation) and with respect to their potential to induce blast-proliferation in a whole blood (WB) culture-system. We studied 3 different markers (IPO-38, Ki-67, CD71) and quantified blast proliferation before/after culture. We correlated findings with (ex vivo) antileukemic functionality, with disease-entities and the course of the disease.

**Results:**

**DC-generation**: We could generate DC/DCleu regularly from WB culture from 36 AML-pts.

**Detection of blast proliferation:** Ø65.6% (range46-82%) of uncultured blasts expressed IPO-38, 33.1% (16-50%) CD71, 25.4% (8-43%) Ki-67.

**Induction of blast proliferation:** Pooling all results we found lowest amounts of proliferating blasts after culture with Kit I (GM-CSF + Picibanil, 10% +/−13.32), Kit K (GM-CSF + PGE2, 9.14% +/−12.01), Kit M (GM-CSF + PGE1, 7.67% +/−11.79). Amounts of proliferating blasts were lower compared to uncultured cells. Highest expression of proliferating blasts was found with IPO-38 followed by CD71 and Ki-67. We found few individual AML-samples with increased blast-proliferation after ex vivo Kit-culture.

**Antileukemic activity:** T-cells stimulated with DCleu (generated with Kits) improved antileukemic activity. Correlations between blast-proliferation and antileukemic activity will be presented.

**Clinical correlations:** Pts with bad (vs good) cytogenetic risk were characterized by higher proportions of proliferating blasts in uncultured blasts; in some Pts with iron-deficiency *anemia* (IDA) proportions of CD71 + uncultured blasts were lower than of IPO-38/Ki-67+ blasts.

**Conclusions:**

IPO-38 is a stable marker to be used to quantify proliferating blasts in AML-pts. CD71 is also a good marker, although not suitable for some pts with IDA, Ki-67 is no reliable marker for every given pt. Subtypes of pts correlated with proportions of proliferating blasts. In general KIT treatment of blasts did only exceptionally induce blast proliferation ex vivo. In general lowest risk for blast proliferation was seen after culture with KIT I, K and M. T-cell-stimulation with DC/DCleu generated after Kit-treatment resulted regularly in antileukemic reactivity. We conclude that an in vivo treatment of AML-pts with Kits I, K or M might be safe (no induction of blast proliferation).

## Tumor Immunity and Microenvironment

### A9 Tumor regression induced by type I interferon triggering in mammary tumor-bearing mice

#### M. Guerin^1,2^, J. M. Weiss^3^, F. Regnier^1,2^, G. Renault^1,2^, L. Vimeux^1,2^, E. Peranzoni^1,2^, V. Feuillet^1,2^, M. Thoreau^1,2^, T. Guilbert^1,2^, A. Trautmann^1,2^, N. Bercovici^1,2^

##### ^1^Cochin Institute, Paris, France; ^2^Inserm U1016 CNRS UMR 8104 Univ Paris Descartes, Paris, France; ^3^Univ Medical Center Freiburg, Freiburg, Germany

###### **Correspondence:** N. Bercovici

**Background:** Although regressing tumors are frequently associated with a large immune infiltrate, the cellular interactions that govern a successful anti tumor immunity remains elusive. Here, we have triggered Interferon-type I signalling in a breast tumor model (MMTV-PyMT) using DMXAA, a ligand of STING (a key detector of cytoplasmic DNA and of viral infection).

**Materials and methods:** Mice with MMTV-PyMT tumors have received a single intraperitoneal injection of DMXAA (23 mg/Kg) and we have examined the dynamics of the immune response which takes place in the tumor microenvironment during tumor regression. The tumor vasculature was visualized by contrast-enhanced ultrasound. Selective depletion experiments using antibody and inhibitor treatments in vivo, together with dynamic imaging, allowed to characterize the cooperation between immune cell subsets.

**Results:** We show that the disruption of the tumor vasculature and the associated IFN alpha/beta release, conditioned a swift and massive recruitment of neutrophils, followed by a delayed rise in CD8 T cells and inflammatory monocytes in the tumor mass. All these immune cell subsets contributed to the efficacy of tumor regression. In particular, we show that neutrophils and monocytes were necessary for an optimal CD8 T cell recruitment and activation. In return, monocytes get activated by infiltrating-CD8 T cells.

**Conclusions:** Altogether, these data highlight that type I interferon signalling is crucial to bridge tumor detection and the initiation of an efficient anti-tumor immune response. We provide evidence for determinant interactions between myeloid cells and T cells that are reminiscent of immune response controlling infections and strongly suggest further developpment of strategies that mobilize and favor cooperations between various immune cell subsets in the tumor.

### A10 A new method to generate mature (leukemia-derived) dendritic cells that improve antileukemic T-cell reactivity from mononuclear cells or whole blood from healthy volunteers or patients with AML

#### D. C. Amberger^1^, F. Doraneh-Gard^1^, C. L. Boeck^1^, C. Plett^1^, C. Gunsilius^1^, C. Kugler^1^, J. Werner^2^, J. Schmohl^2^, D. Kraemer^3^, B. Ismann^4^, A. Rank^5^, C. Schmid^5^, H. M. Schmetzer^1^

##### ^1^University Hospital of LMU, Department for Hematopoietic cell transplantation, Med3, Munich, Germany; ^2^University Hospital of Tuebingen, Department for Hematology and Oncology, Tuebingen, Germany; ^3^University Hospital of Oldenburg, Department for Hematology and Oncology, Oldenburg, Germany; ^4^University Hospital of LMU, Department for Hematology and Oncology, Med3, Munich, Germany; ^5^Municipal Hospital of Augsburg, Department of Hematology and Oncology, Augsburg, Germany

###### **Correspondence:** D. C. Amberger

**Background:** Dendritic cells (DC) and ‘leukemia-derived DC’ (DC_leu_) are potent stimulators of various immunoreactive cells and play a pivotal role in the (re-)activation of the immune system.

**Materials and methods:** We established a new protocol, containing *GM-CSF, IL-4, Picibanil* and *PGE*_*1*_ (**‘Pici2’**), to generate DC/DC_leu_ ex-vivo from mononuclear cells (**‘MNC-DC’**) or directly from whole blood (to simulate in-vivo conditions, **‘WB-DC’**) from AML-patients. Further, we analysed the DC-generating-potential of the **‘IMMUNOMODULATORY KIT-M’** containing *GM-CSF* and *PGE*_*1*_. Results with regard to DC-, T-cell-subtypes and mediation of antileukemic activity were compared to an established DC-generating protocol (‘Pici1’ containing GM-CSF, IL-4, Picibanil, PGE_2_) or to a culture without cytokines.

**Results:** **1.) DC-generation** **:** *healthy-‘MNC/WB-DC’:* We generated comparable DC-proportions with ‘Pici2’ and the standard protocol ‘Pici1’. With ‘Pici2’ we significantly (p < 0.001) improved the DC-amounts compared to controls without cytokines. *AML-‘MNC-DC’:* In contrast to cases with ‘Pici1’, ‘Pici2’ performed a sufficient DC-generation (≥10% DC) in every case. The amounts of DC_leu_ were comparable with both protocols. *AML-‘WB-DC’* : ‘Kit-M’ enabled a sufficient DC-generation in >65% of cases. Further, we generated higher amounts of DC_leu_ with the two PGE_1_ containing ‘cocktails’ (DC_leu_: range: 3.4%-13.6%) in comparison to controls without cytokines. **2.) Mature DC:** Adding PGE_1_ to the ‘DC-generating-cocktails’ increased the proportions of mature DC (CD197^+^) in comparison to ‘Pici1’ in AML-samples. This effect, however, did not occur in healthy-samples. **3.) T-cell-subtypes:** T-cell-stimulation with DC, generated with PGE_1_ containing ‘cocktails’, positively influenced the composition of T-cell-subsets, characterised by higher proportions of proliferating (CD71^+^), non-naïve (CD45RO^+^), central-(memory) (CD45RO^+^CCR7^+^) and effector-(memory)T-cells (CD45RO^+^CCR7^−^). **4.) Antileukemic activity:** T-cells stimulated with DC, generated with ‘Kit-M’ and ‘Pici2’ highly improved antileukemic activity (‘blast lysis’) in >70% of cases.

**Conclusions:** We developed a new DC/DC_leu_ generating protocol and demonstrated that PGE_1_ is suitable for generating DC/DC_leu_ in sufficient amounts. These cells reliably (re-)activate immunoreactive cells and improve the overall ex-vivo antileukemic activity. In-vivo trials (animals and/or humans) have to be performed to study potential effects of PGE_1_ in the mediation of antileukemic reactions in-vivo in patients with AML.

### A11 Interleukin-22 (IL-22) does not influence methycholanthren-A-mediated tumorigenesis

#### A. Markota, C. Ochs, P. May, A. Gottschlich, J. Suárez Gosálvez, C. Karches, D. Wenk, S. Endres, S. Kobold

##### Divison of Clinical Pharmacology, Department of Internal Medicine IV, Ludwig-Maximilians Universität, Munich, Germany

###### **Correspondence:** A. Markota

**Background:** Interleukin-22 (IL-22) is a unique cytokine expressed by immune cell subtypes such as T and NK cells and acting exclusively on Interleukin-22-receptor-1 (IL-22-R1) positive epithelial cells. Recently, we have demonstrated that expression of IL-22 is frequently found in primary human small and large cell lung cancer samples. It is also known that IL-22 is found at high levels in the primary tumor, serum and in the malignant pleural effusion of non-small cell lung cancer patients. However, the influence of IL-22 on the development of a spontaneous experimental tumor remained unaddressed.

**Materials and methods:** To test the influence of IL-22 on tumorigenesis, we used the murine methylcholantren-A (MCA) model. MCA was injected at different doses (100 μg, 200 μg and 400 μg) subcutaneously in wild type or IL-22-KO Balb/c mice. Tumor onset and growth was monitored.

**Results:** The time of tumor development correlated with the dose of MCA used. In wild type mice, 100, 200 and 400 μg of MCA led to a median onset of tumor growth (first day of palpable lesion) at days 124, 83 and 70 respectively (n = 4 to 6 per group). IL-22-KO mice had similar intervals until tumor onset of 131, 88 and 70 days, respectively. Tumor growth after development was also similar between MCA dosage groups. No difference in median overall survival was noted in IL-22-KO (173, 161 and 164 days) versus wild type (163, 165 and 165 days) mice. We could not observe any impact of IL-22-KO on the phenotype of mice developing sarcoma (weight or behavior).

**Conclusions:** Endogenous IL-22 does not appear to play a role in MCA-induced sarcoma model in Balb/c mice. The findings in this inflammation-based tumorigenesis model are in contrast to published findings on enhancing tumor promoting role of IL-22 in the APC-min colon cancer model.

### A12 Inhibition of CD39 and CD73 by 3rd generation antisense oligonucleotides to improve immunity against tumors

#### T. Hilmenyuk, R. Klar, F. Jaschinski

##### Secarna Pharmaceuticals GmbH & Co. KG, Planegg/Martinsried, Germany

###### **Correspondence:** T. Hilmenyuk

**Background:** During the last decades it became obvious that the immune system can be utilized to evoke effective anti-tumor responses. However, cancer cells develop mechanisms to circumvent this. The two ectonucleotidases CD39 and CD73 are promising drug targets, as they act in concert to convert extracellular immune-stimulating ATP to immunosuppressive Adenosine. CD39 and CD73 are expressed on different immune cells as well as on cancer cells. Accordingly, in order to enhance immunity against tumors it would be favorable to increase extracellular ATP- and to reduce Adenosine concentrations in the tumor microenvironment.

**Materials and methods:** We designed unformulated antisense oligonucleotides (ASOs) with specificity for human CD39 (hCD39) and CD73 (hCD73). ASOs were synthesized as GapmeRs with flanking locked nucleic acid modifications to increase stability and affinity to the target RNA. Knockdown efficacy of ASOs on mRNA and protein level was investigated in cancer cell lines and primary human T cells. CD39 activity was evaluated by measuring ATP degradation in cell supernatants. We determined CD73 activity by quantification of free phosphate, released during the conversion of AMP to Adenosine. As functional readout we analyzed T cell proliferation and viability in presence of extracellular ATP and Adenosine.

**Results:** Our initial experiments revealed, that CD39 was predominantly expressed on activated CD8^+^ T cells whereas CD73 was exclusively expressed on activated CD4^+^ T cells, suggesting that both T cell populations work in concert to convert immune stimulating ATP to immune suppressive adenosine. Furthermore, we investigated a successful knockdown of CD39 and CD73 on mRNA and protein level in cancer cell lines and primary CD4^+^ and CD8^+^ T cells after administering ASOs. Moreover, enzymatic activity of CD39 and CD73 was blocked by ASO treatment. Addition of extracellular ATP significantly impaired proliferation and viability of CD39 expressing CD8^+^ T cells probably via the formation of immunosuppressive ATP-degradation products. Strikingly, administration with CD39-ASO but not with unspecific control ASO successfully reversed this inhibition. Extracellular AMP strongly inhibited proliferation and viability of CD73-competent CD4^+^ T cells most likely by the generation of immunosuppressive Adenosine. Notably, CD73-ASO treatment protected CD4^+^ T cells from proliferative inhibition. Our initial *in vivo* studies in mice applying mouse specific CD39-ASOs systemically demonstrated a successful knockdown of CD39 mRNA expression in spleens and lymph nodes. The impact of CD39- and CD73-ASO knockdown *in vivo* on anti-tumor immune responses, tumor growth and survival will be subsequently analyzed in forthcoming studies.

**Conclusions:** Therefore, targeting CD39 and CD73 by ASOs represents a very promising state-of-the art therapeutic approach to improve immunity against tumors.

### A13 The immunome in early stage NSCLC: flow cytometry analyses of immunological subpopulations in tumor, lung tissue and whole blood

#### G. Gamerith^1^, F. Augustin^2^, E. Lorenz^3^, C. Manzl^4^, E. Hoflehner^1^, P. Moser^4^, B. Zelger^4^, S. Köck^1^, A. Amann^1^, J. Kern^3^, G. Schäfer^4^, D. Öfner^2^, H. Maier^2^, H. Zwierzina^1^, S. Sopper^1^

##### ^1^Clinic of Internal Medicine V, Medical University of Innsbruck, Innsbruck, Austria; ^2^Department of Visceral, Transplant and Thoracic Surgery, Medical University of Innsbruck, Innsbruck, Austria; ^3^Tyrolean Cancer Research Institute, Innsbruck, Austria; ^4^Department of Pathology, Medical University of Innsbruck, Innsbruck, Austria

###### **Correspondence:** G. Gamerith

**Background:** Immune checkpoint therapies have recently revolutionized cancer treatment highlighting the crucial role of the immune system in cancer. The prognosis and outcome is based on subtype, location and function of various immune cells, including T-lymphocytes, dendritic cells, but also the innate immune system. To define patients with a high probability for response and thus improve outcome and cost effectiveness, a proper knowledge of immunological alterations is highly warranted. We therefore investigate the immunome of early stage NSCLC patients in a prospective setting.

**Materials and methods:** Approval by the regional ethics board and ICs were given. Whole blood (WB), tumor and adjacent normal tissue of 40 early stage NSCLC patients were collected and single cells suspension achieved via mechanical disruption and enzymatic digestion. To characterize the cellular composition, differentiation and activation status of immune cells, 9 color flow cytometry was performed using six antibody panels aimed at major leukocyte populations. Samples were analyzed on a FACS Fortessa using FlowJo software. Significance (p < 0.05) was determined by Friedman tests with Dunn’s multiple comparison analyses.

**Results:** Granulocytes were less frequent in tumor and normal tissue compared to WB. As a consequence, proportion of lymphocytes among leucocytes was significantly higher in lung and tumor tissues. Regarding lymphocyte subsets, proportions of T cells and the major CD4-, CD8- and γδTCR-expressing subsets were comparable in all three compartments whereas B cells tended to be higher in tumor and lower in lung tissue. Vice versa, proportion of NK cells was significantly lower in tumor compared to normal tissue and WB, accompanied by a lower CD158b (KIR2D) expression. In contrast, the inhibitory receptor CD159a (NKG2A) was expressed in a higher proportion in the tumor compared to both blood and healthy tissue. Naïve T cells were nearly absent in lung and tumor tissue. CD4 T cells in tumor were enriched for central memory cells, whereas in healthy tissue effector memory cells prevailed. Compared to blood a higher proportion of CD8 T cells expressed the early activation marker CD69 in normal tissue and even higher in tumors. The chemokine receptor CD183 (CXCR3) was expressed in tumor on a significantly larger subset of B cells than in WB and lung tissue, indicating its involvement in the regulation of migration of B cells to the tumor site. Our results underline changes in the immunome to occur early in lung tumorgenesis and highlight the importance of NK and B cell subpopulations. Therefore in addition to immune checkpoint inhibition drugs that also target B cells and the innate immune system are required.

**Conclusions:** We demonstrate changes in the immunome in early stage NSCLC patients and found NK and B cells to be the most altered. Further investigations concerning the functional and prognostic impact of our results are warranted.

### A14 Mitochondrial mass analysis in CD8+ T cells from lung cancer patients

#### H. Prado-Garcia, S. Romero-Garcia, R. Sandoval-Martínez, A. Puerto-Aquino, J. Lopez-Gonzalez, U. Rumbo-Nava

##### National Institute of Respiratory Diseases, Mexico City, Mexico

###### **Correspondence:** H. Prado-Garcia

**Background:** CD8+ T cells undergo major metabolic changes upon activation, but how mitochondrial metabolism is altered in lung cancer is still unknown. Malignant pleural effusions, which are infiltrated by lymphocytes and tumor cells, are frequent in patients with advanced stages of lung cancer. Pleural effusion CD8+ T cells from lung cancer patients show reduced effector functions which might be caused by a deficient mitochondrial response.

**Materials and methods:** Pleural effusion or peripheral blood CD4+ and CD8+ T cells, from lung cancer patients and healthy donors, were stimulated with anti-CD3 antibody or bead-coated anti-CD3 and anti-CD28 antibodies for 24 h under normoxic and hypoxic (2%O_2_) conditions. Then, mitochondrial mass was analyzed using MitoTracker Green on CD8+ T cells by flow cytometry. Also, Nuclear Respiratory Factor-1 (NRF-1) protein levels were measured on purified CD8+ T cells by immuno-PCR after anti-CD3 stimulation.

**Results:** Under non-stimulated conditions, mitochondrial mass, measured as Mean Fluorescence Intensity of Mitotracker Green, was higher in pleural effusion CD8+ T cells compared with that of peripheral blood CD8+ T cells from cancer patients and healthy donors. After stimulation with anti-CD3 and anti-CD28 antibodies, memory CD45RA-CD27 + CD8+ T cells from healthy donors reduced their mitochondrial mass; this phenomenon was not observed in memory CD8+ T cells from lung cancer patients. Moreover, after anti-CD3 stimulation under hypoxic conditions, memory CD8+ T cells from peripheral blood reduced their mitochondrial content, in both cancer patients and healthy donors. By contrast, memory CD8+ T cells from malignant effusions were not able to reduce their mitochondrial mass. In addition, NRF-1 levels were reduced in pleural effusion CD8+ T cells compared to those from peripheral blood from cancer patients and healthy donors. After anti-CD3 stimulation, NRF-1 levels were increased in peripheral blood but not pleural effusion CD8+ T cells from lung cancer patients.

**Conclusions:** Pleural effusion CD8+ T cells from lung cancer patients did not modify their mitochondrial content and NRF-1 levels in response to activation or hypoxia, which might compromise the metabolism and function of these cells.

### A15 Targeting hIDO1 with 3rd generation antisense oligonucleotides for modulation of the tumor microenvironment

#### R. Klar, T. Hilmenyuk, F. Jaschinski

##### Secarna pharmaceuticals GmbH & Co KG, Martinsried, Germany

###### **Correspondence:** R. Klar

**Background:** Targeting the immunosuppressive shield of tumors has emerged as a promising treatment option for oncologic indications in the last years. Especially the success of antibodies targeting CTLA-4 and PD-1 in late stage melanoma patients paved the way for immuno-oncology in daily clinical practice. However, despite long lasting remissions in a small subset of tumor patients the majority of patients does not respond to the currently available immunotherapies. This is based on the fact that tumor cells and suppressive immune cells can express a plethora of immunosuppressive factors leading to immune evasion of the tumor. One of those factors is the enzyme indoleamin-2,3-dioxygenase 1 (IDO1) which is a critical factor in the metabolism of tryptophan. The degradation of tryptophan in the tumor microenvironment leads on the one hand to tryptophan starvation and on the other hand to the generation of kynurenines both resulting in a suppression of immune effector cells.

**Materials and methods:** We designed antisense oligonucleotides (ASOs) with specificity for human IDO1 (hIDO1). ASOs were synthesized as GapmeRs with flanking locked nucleic acids to increase stability and affinity to the target RNA. The knockdown efficacy of ASOs on the mRNA and protein level was investigated in cancer cell lines and primary human cells after ASO treatment without addition of a transfection reagent. The effect of hIDO1 knockdown on the production of L-Kynurenine was determined by measurement of the L-Kynurenine concentration in cell culture supernatants by ELISA.

**Results:** We tested the mRNA knockdown efficacy of a number of ASOs in two cancer cell lines, namely EFO-21 and SKOV-3. Strikingly, a subset of ASOs resulted in a hIDO1 mRNA knockdown of >90%. The IC50 values were determined in EFO-21 cells which resulted in the selection of two highly potent ASOs with IC50 values in the low nanomolar range for further experiments. The treatment of cancer cell lines as well as primary human cells resulted in reduction of IDO protein levels by >85%. Importantly, we observed a complete block in the production of immunosuppressive L-Kynurenine in ASO treated cells and IC50 values in the low nanomolar range.

**Conclusions:** We designed highly potent hIDO1 ASOs that efficiently knock down hIDO1 mRNA and protein and block the production of immunosuppressive kynurenines in cancer cell lines as well as primary human cells. We are furthermore investigating the effect of hIDO1 knockdown in cancer cell lines and dendritic cells on the function of T cells and NK cells *in vitro* at the moment. Experiments proving the efficacy of IDO1 ASOs in syngeneic tumor models are ongoing using mouse-specific ASOs. Taken together, we developed an innovative immunotherapeutic tool to block the expression of hIDO1 that will potentially improve treatment options for cancer patients in the future.

### A16 Relevance of systemic immunosuppression in ovarian cancer patients

#### A. Coosemans^1,2^, T. Baert^1,2^, A. Van Hoylandt^1^, P. Busschaert^3^, I. Vergote^3,2^

##### ^1^KU Leuven, Department of Oncology, Laboratory of Tumor Immunology and Immunotherapy, Immunovar Research Group, Leuven, Belgium; ^2^UZ Leuven, Gynaecology and Obstetrics, Leuven Cancer Institute, Leuven, Belgium; ^3^KU Leuven, Department of Oncology, Laboratory of Gynaecologic Oncology, Leuven, Belgium

**Background:** Ovarian cancer is a silent killer, metastasising throughout the abdomen before causing symptoms. This immediately explains that mortality (which is the case in 80% of patients) is caused by the metastases. So far, research concerning the immune system in ovarian cancer has focussed on the tumor microenvironment. The peripheral immune landscape in ovarian cancer remains largely undiscovered.

**Materials and methods:** Peripheral blood mononuclear cells were collected prospectively in 39 patients with invasive ovarian cancer. Samples were prelevated at diagnosis, after cytoreductive surgery ((interval-)debulking), after three courses of (neo-)adjuvant platin-based chemotherapy, and at the end of their primary treatment. Fluorescent activated cell sorting was applied to detect immunostimulatory cells (T_helper_ cells (CD4^+^), T_cytotoxic_ cells (CD8^+^), natural killer cells (NK)) and immunosuppressive cells (regulatory T cells (Treg), mMDSC (monocytic myeloid-derived suppressor cells), gMDSC (granulocytic MDSC)).

**Results:** Patients were distributed as follows: 76% had an advanced stage of the disease at diagnosis (stage III and IV), 44% relapsed, including six deaths. Tumors had a high-grade histology in 80% of cases. Stage IV ovarian cancers presented with higher amounts of mMDSC (p 0.007). High-grade ovarian cancers presented with more PD1^+^MDSC (p 0.03) (PD1 = programmed cell death protein 1). After primary treatment (i.e. chemotherapy and debulking surgery) there was an increase of T_cytotoxic_ cells (p 0.002) and activated T_cytotoxic_ cells (CD8^+^CD69^+^)(p 0.02). Debulking surgery increased the number of activated T_helper_ cells (CD4^+^CD69^+^)(p 0.02). Progression free survival was significantly reduced in case of increasing amount of T_helper_ cells (p 0.02), activated T_helper_ cells (p 0.002), activated T_cytotoxic_ cells (p 0.01), mMDSC (p 0.03) and PD1^+^mMDSC (p 0.002). Overall survival was reduced in case of increasing activated T_helper_ cells (p 0.04) and activated T_cytotoxic_ cells (p 0.04).

**Conclusions:** In contrast to what is seen in literature in the primary tumor, a high presence of T_helper_ cells and T_cytotoxic_ cells in the systemic circulation of ovarian cancer patients is correlated with a worse survival. It almost seems that the systemic immune system represents a state of immune alert of the body as a reaction to widespread metastatic disease but that this is insufficient. mMDSC seem to be an important immunosuppressive player in ovarian cancer.

The authors would like to thank the Olivia Hendrickx Research Foundation for their support.

### A17 Monocytic myeloid derived suppressor cells (mMDSC) determine outcome in ovarian cancer

#### T. Baert^1,2^, A. Van Hoylandt^1^, P. Busschaert^3^, I. Vergote^3,2^, A. Coosemans^1,2^

##### ^1^KU Leuven, Department of Oncology, Laboratory of Tumor Immunology and Immunotherapy, Immunovar Research Group, Leuven, Belgium; ^2^UZ Leuven, Gynaecology and Obstetrics, Leuven Cancer Institute, Leuven, Belgium; ^3^KU Leuven, Department of Oncology, Laboratory of Gynaecologic Oncology, Leuven, Belgium

###### **Correspondence:** T. Baert

**Background:** Ovarian cancer is considered a highly aggressive tumour, killing still 80% of patients. So far immunotherapy trials have yielded poor results, due to severe immunosuppression. Myeloid derived suppressor cells (MDSC) are a heterogeneous group of immature myeloid cells that have a strong immunosuppressive function. MDSC can be subdivided into monocytic MDSC (mMDSC) and granulocytic MDSC (gMDSC) based on the relative surface expression of lymphocyte antigen 6 complex locus C (Ly6C) and Ly6G in mice and cluster of differentiation 14 (CD14) and CD15 in humans.

**Materials and methods:** The murine experiments were performed in C57BL/6 mice (n = 30) and Rag1^*tm1Mom*^ mice (n = 12), inoculated intraperitoneal (ip) with 5 × 10^6^ ID8-fLuc cells. Mice received ip injections with phosphate buffered saline (PBS), PBS liposomes or clodronate liposomes twice weekly starting from tumor inoculation. Six mice per group were evaluated for survival analysis. Three and four weeks after tumor challenge three mice per group were sacrificed for immune-monitoring on ascites and serum. Immune cells were characterized by fluorescent activated cell sorting (FACS), cytokines were determined by cytometric bead array (CBA). Next, peripheral blood mononuclear cells were collected prospectively in 39 ovarian cancer patients at diagnosis. MDSC were defined by FACS as live CD11b^+^HLA-DR^−^ cells.

**Results:** Mice treated with clodronate liposomes died significantly faster compared to immunocompetent mice (p = 0.004), whereas the survival was not altered in mice lacking T- and B-cells (Rag1^*tm1Mom*^). After treatment with clodronate liposomes we observed a significant decrease in macrophages (p = 0.001). mMDSC (live CD11B^+^Ly6C^Hi^) increased significantly (p = 0.004) after treatment with clodronate liposomes. In patient samples, we observed a significant decrease in progression free survival in patients with a high number of CD14^+^ mMDSC (p = 0.03).

**Conclusions:** The administration of clodronate liposomes significantly reduced survival of mice with ovarian cancer, most likely due to a depletion of macrophages and a significant increase in mMDSC. This is in accordance with the situation in ovarian cancer patients, where increasing mMDSC are correlated with decreasing progression free survival.

The authors would like to thank the Olivia Hendrickx Research Fund for their support.

### A18 GBP1 expression in colorectal cancer liver metastasis associates with increased CD8+ lymphocyte invasion and confers better prognosis

#### J. Laengle

##### Department of Surgery, Vienna, Austria

**Background:** Preclinical models indicated that both, type I and type II IFN, response gene expressions are relevant for the induction of a tumor-ablative cytotoxic T cell induction. Furthermore, GBP1 expression in primary colorectal tumor cells was previously shown to be a prognostic factor. Therefore, we investigated the correlation between GBP1 and MxA in colorectal liver metastasis (CRLM), tumor infiltrating lymphocytes and recurrence free survival after surgical resection and overall survival.

**Materials and methods:** 24 colorectal cancer patients with surgically treated liver metastasis were included in the study between 2005 and 2011. Immunohistochemical labeling of GBP1, MxA, Ki67, CD45RO, CD3 and CD8 was performed on formalin-fixed paraffin-embedded histological sections. Stainings were semiquantitatively scored for both tumor specific and stromal expression. Median follow-up time was 1300 days.

**Results:** Tumor cell specific GBP1 expression was present in 63% of the cases. The majority of tumors displayed MxA positivity to a varying degree. Intratumoral infiltration of CD3+ or CD8+ lymphocytes was found in 16% and 26% of tumors, respectively. Importantly, both CD3+ and CD8+ lymphocytic infiltration was associated with GBP1 expressing tumor cells of CRLM. GBP1 positive tumors tended to have a lower Ki-67 labeling index, however, Ki-67 showed no prognostic power for overall survival. In contrast, GBP1 expression was a significant prognosticator for overall survival and showed a tendency for predicting recurrence-free survival. MxA expression did not show prognostic power or correlation with infiltrating lymphocytes. The presence of intraepithelial CD8+ tumor infiltrating lymphocytes was mostly found in long-term surviving patients.

**Conclusions:** Tumor cell specific GBP1 expression in CRLM is associated with CD3+ and CD8+ lymphocytic infiltration and with overall survival. Thus, we propose that in future clinical studies exploring novel immunotherapeutic approaches in colorectal cancer the GBP1 expression should also be evaluated.

### A19 Circulating myeloid-derived suppressor cells (MDSC) are increased in breast and colorectal cancer patients

#### K. Pilatova^1,2^, E. Budinska^1,3^, B. Bencsikova^1,2^, R. Sefr^1,2^, R. Nenutil^1^, V. Brychtova^1^, L. Fedorova^1,2^, B. Hanakova^1,3^, L. Zdrazilova-Dubska^1,2^

##### ^1^Masaryk Memorial Cancer Institute, Brno, Czech Republic; ^2^Faculty of Medicine Masaryk University, Brno, Czech Republic; ^3^Faculty of Science Masaryk University, Brno, Czech Republic

###### **Correspondence:** L. Zdrazilova-Dubska

**Background:** Myeloid-derived suppressor cells are heterogenic population of immunosuppressive cells of myeloid lineage. Recently, MDSC were studied in the context of tumour growth, where they are responsible for the tumour escape from the host immune surveillance via the mechanism of inhibition of CD8+ T cells and by promotion of angiogenesis and metastatic spread. In several types of cancer, increased number of MDSC correlated with worse prognosis and mortality. MDSC can be classified as granulocytic (Gr-MDSC) and monocytic (Mo-MDSC). Here we evaluate circulating Mo-MDSC in non-cancer individuals and examined whether Mo-MDSC are increased in breast and colorectal cancer patients.

**Materials and methods:** We measured Mo-MDSC counts in peripheral blood of non-cancer individuals representing the control group (N = 61), and breast cancer (N = 39) and colorectal cancer (N = 52) patients. MDSC were detected as CD45 + CD11b + CD33 + CD14 + HLA-Dr^low/-^ and quantified as proportion (%) of MDSC in total white blood cells and as an absolute count. Two-sample two-tailed Mann–Whitney *U* Test was used to examine differences in Mo-MDSC between control and cancer groups and Wilcoxon signed-rank test was used in paired sample comparison of the Mo-MDSC at the time of diagnosis vs at the time of follow-up.

**Results:** In the control group, the Mo-MDSC count was gender- and age-independent, with the average of 0.073 × 10^9^/l and 1.09%. Breast cancer patients, both count and proportion of circulating Mo-MDSC was significantly higher (p < 0.001) compared to the control group with average levels of 0.229 × 10^9^/l and 3.57%, respectively. In breast cancer patients, we observed increase in Mo-MDSC number after the administration of granulopoietic growth factors. Compared to control group, colorectal cancer (CRC) patients had significantly higher count and proportion of circulating Mo-MDSC with average values of 0.125 × 10^9^/l, 1.71%. Number of Mo-MDSC did not correlate with tumour clinicopathologic stage, localization (colon vs. rectum), side (left vs. right) and microsatellite instability (MSI). During 6-month follow-up of 19 CRC patients, we observed increase in Mo-MDSC absolute counts and proportion in 74% patients, and decrease in 11% patients.

**Conclusions:** Colorectal cancer patients at diagnosis showed higher counts of circulating Mo-MDSCs, possibly due to immunosuppressive effect of the tumour microenvironment. The change in Mo-MDSC count from baseline needs to be evaluated in the context of CRC patient outcome. Recombinant granulopoietic growth factors increase number of circulating Mo-MDSCs and the effect of this phenomenon on breast cancer prognosis remains to be elucidated.

Supported by Ministry of Health of Czech Republic via grant nr.16-31966A and by MEYS-NPS I - LO1413.

### A20 Gene expression profile of the tumor microenvironment from 40 NSCLC FFPE and matched fresh frozen samples using a targeted NGS solution

#### Chris Allen, Yuan-Chieh Ku, Warren Tom, Yongming Sun, Alex Pankov, Tim Looney, Fiona Hyland, Janice Au-Young, Ann Mongan

##### Thermofisher Scientific, South San Francisco, CA, United States

###### **Correspondence:** Chris Allen

**Background:** The tumor microenvironment (TME) is the intersection between tumor cells and surrounding non-transformed cells. It contains immune cells, signaling molecules, stromal and extracellular matrix. The tumor microenvironment is often promoting tumor growth during all stages of carcinogenesis. However, the function and regulatory mechanism of each constituent is still poorly understood. Recent studies show that T cell checkpoint therapy provides durable and therapeutic results for non-small cell lung cancer (NSCLC). The presence of PD-L1 is a promising marker to predict positive response. Current IHC methods to measure PD-L1 are subjective and highly variable. A higher-throughput and standardized method that can systematically measure gene expression of cells present in the TME has emerged to be a more desirable solution.

**Materials and methods:** We applied the Oncomine^TM^ Immune Response Research Assay to measure the expression of 395 genes in NSCLC research samples from 40 matched FFPE and fresh frozen sample types. This assay covers genes involved in checkpoint pathway, T cell regulation, cytokine and interferon signaling pathways, and markers of different tumor infiltrating lymphocyte TIL subsets, as well as tumor markers. With an input requirement of 10 ng of total RNA, libraries were generated, templated on the Ion Chef^TM^ and sequenced on the Ion S5^TM^ System. Sequencing data was analyzed and mapped with Torrent Suite Software and differential expression analysis was conducted with Affymetrix^TM^ Transcriptome Analysis Console.

**Results:** Our data showed that gene expression profiles can stratify NSCLC FFPE samples by histopathology and TIL levels. The expression profiles of FFPE and fresh frozen samples are highly correlated with an average correlation greater than 0.9. Approximately 80% of the 40 samples show moderate to high expression of CD8+ T cell cytokines, IFNG and TNFa. We conducted a differential expression analysis between a group of samples (n = 8) with high percentage of surrounding and infiltrating lymphocytes and another group (n = 5) with low stromal content but devoid of infiltrating lymphocytes. We found a large number of genes which annotated as infiltrating lymphocyte markers (CTSS, CXCR4, CD37, SRGN, FCER1G, SAMHD1, and GZMA) are significantly up-regulated in samples with high percentage of surrounding and infiltrating lymphocytes. The expression of 22 genes in the panel was validated with qPCR using FFPE samples. These genes cover a range of low, medium, and high expressors according to our NGS data. There is a strong correlation (R > 0.9) between NGS and qPCR data.

**Conclusions:** In summary, this study highlights the robustness of using a targeted panel to understand the composition and regulatory mechanism of the TME and tumor immune response. For research use only. Not for use in diagnostic procedures.

### A21 Small-molecule inhibitors of ecto-nucleotidase CD73 result in strong activation of human and mouse CD8^+^ T cells in the presence of exogenous AMP, affect tumor growth and positively affect anti-tumor immunity in mouse syngeneic tumor models

#### A. Becker, J. B. L. Tan, A. Chen, K. Lawson, E. Lindsey, J. P. Powers, M. Walters, U. Schindler, S. Young, J. C. Jaen

##### ARCUS Biosciences, Hayward, CA, United States

###### **Correspondence:** A. Becker

**Background:** Adenosine (ADO), apotent inhibitor of T-cell activation, is generated within the tumor through coordinated and sequential cleavage of extracellular adenosine triphosphate (ATP) by the ecto-nucleotidases CD39 (which produces adenosine monophosphate, AMP) and CD73 (which hydrolyzes AMP to ADO). Whereas a number of anti-CD73 antibodies are being advanced into clinical trials, to date there have been only a few reports of potent and selective small molecule CD73 inhibitors, such as those described here.

**Materials and methods:** *Enzymatic assays:*Ecto-nucleotidase activity was determined by calculating the amount of inorganic phosphate (malachite green assay; absorbance at 620 nm) produced after 50-min incubation with 25 μM AMP or ATP, in the presence of varying concentrations of test compound(s). The following systems were used. Endogenous expression: hCD73/SKOV-3 cells; mCD73/E771 cells. Stable over-expression: hCD73/CHO; hCD39/CHO. Transient expression: mCD73/CHO; NTPDase2/CHO; NTPDase3/CHO; NTPDase8/CHO. *Immune Function Assays: CD8* ^*+*^ *T cell assays:* Human CD8^+^ T cells were isolated from buffy coats and pretreated with CD73 inhibitor or a control compound for one hour. Afterwards, the cells were activated (anti-CD2/CD3/CD28) in the presence of 50 μM AMP and 10 μM ADO deaminase inhibitor (EHNA). In some experiments, exogenous recombinant human IL12p70 was added to the culture. Following 3 days, activation (CD25) and effector functions (Granzyme B and IFNγ) were measured by flow cytometry. *Tumor model:* CT26 and B16F10 cells were subcutaneously implanted into 8 week old Balb/c or C57BL6J mice, respectively. After tumor volumes reached ~60-150 mm^3^, therapeutic dosing using 30 mg/kg A000830 s.c., q.d. was initiated. αPD1 was dosed at 5 mg/kg (B16F10) or 10 mg/kg (CT26) q3d. To analyze tumor infiltrating leukocytes, single cell suspensions were generated from tissues, blocked and stained with the appropriate antibody cocktails.

**Results:** A series of potent and specific small-molecule inhibitors of human and mouse CD73 has been generated, represented by A000830 and A001421, that inhibit the enzymatic activity of human and mouse CD73 with high selectivity against a large panel of related and unrelated enzymes. In an *in vitro* human CD8^+^ T cell activation assay employing primary human and mouse CD8^+^ T cells, both A000830 and A001421 robustly reversed ADO mediated inhibition of CD25 expression as well as production of Granzyme B and IFNγ. Finally, *in vivo* dosing of A000830 in a monotherapeutic setting resulted in limited tumor growth in a CT26 model and to an increased therapeutic effect of αPD1 blocking antibody in a B16F10 model. Tumor growth inhibition in the presence of A00830 and α-PD1 was likely due to increased intra-tumoral ratios of CD8^+^ T cell to T_reg_ or MDSC.

**Conclusions:** We describe a novel class of potent and selective small-molecule CD73 inhibitors that block the generation of ADO from extracellular AMP, restore ADO-mediated inhibition of human and mouse T-cell activation in vitro, and display promising anti-tumor activity in vivo.

### A22 Aberrant status of glutathione metabolism predict prognosis of colorectal cancer patients

#### S. Yin, Y. Chen

##### Medizinische Klinik und Poliklinik IV, Klinikum der Universität München, Muenchen, Germany

###### **Correspondence:** S. Yin

**Background:** Colorectal cancer (CRC) is one of the most frequently diagnosed cancers worldwide. The number of the new cases, especially in developing country, is predicted to climb over the following decades. Altered cancer metabolism has been shown to promote sustained tumorgenesis and the survival of cancer cells under extreme environment. Glutathione represents an important source of energy for tumor cell metabolism. Thus, we hypothesize that the tumor metabolic phenotype of glutathione could be prognosistic factor in CRC patients.

**Materials and methods:** We retrospectively investigated 120 pairs of cancerous and matched noncancerous tissue specimens from CRC patients who underwent the same standard chemotherapy and radical surgery. We stained the successive paraffin-embedded slides for those marker involved in glutathione metabolism (GSTM3, GSTP1, SRM, ODC1) respectively and correlate the expression of these markers with tumor grade, stage, overall survival (OS) and progression-free survival (PFS). We further integrated transcriptome data of 345 CRC patients from public geo database and investigated the correlations.

**Results:** Expression of GSTM3 in tumor group are decreased than in matched noncancerous group, while GSTP1, SRM and ODC1 are overexpressed in tumor group. Moreover, within the tumor group, combined higher expression of GSTP1, SRM and ODC1 had lower 5-year PFS (52%) and OS (58%) than those with lower GSTP1 tumor group (60% and 66%, respectively; PFS HR: 1.89,95% confidence interval(CI): 1.21-3.40, *P* = 0.019; OS HR: 2.34, 95% CI: 1.38-4.32, P = 0.021). While, higher expression group of GSTM3 within tumor group has higher 5-year PFS (70%) and OS (72%) than those with lower GSTP1 tumor group (65% and 66%, respectively; PFS HR: 2.1,95% confidence interval(CI): 1.4-3.21, P = 0.0059; OS HR: 2.03, 95% CI: 1.32-4.17, P = 0.021).

**Conclusions:** The expression pattern of these marker are also correlated with OS and PFS of the patients. Thus, enhanced status of glutathione metabolism could be a useful prognostic factor in CRC patients.

### A23 Gastric cancer with lymphoid stroma: a study of clinico-pathological profile, Epstein-Barr virus, microsatellite instability, tumour immune microenvironment and PD-L1 *status*

#### I. Gullo^1,2,3^, G. Gonçalves^4,1^, M. L. Pinto^1,5,6^, M. Athelogou^7^, G. Almeida^1^, R. Huss^7^, C. Oliveira^1,2^, F. Carneiro^1,8,3^

##### ^1^Institute of Molecular Pathology and Immunology at the University of Porto (Ipatimup) and Institute for Research Innovation in Health (i3S), Porto, Portugal; ^2^Department of Pathology and Oncology, Faculty of Medicine of the University of Porto, Porto, Portugal; ^3^Centro Hospitalar de São João, Porto, Portugal; ^4^Department of Biomedical Sciences and Medicine, University of Algarve, Algarve, Portugal; ^5^INEB-Institute of Biomedical Engineering, University of Porto, Porto, Portugal; ^6^ICBAS-Institute of Biomedical Sciences Abel Salazar, University of Porto, Porto, Portugal; ^7^Definiens AG, Munich, Germany; ^8^Department of Pathology and Oncology, Faculty of Medicine of the University of Porto, Porto, Portugal, Porto, Portugal

###### **Correspondence:** I. Gullo

**Background:** Targeted immunotherapies (TIs) can selectively impair the cancer evasion of immune surveillance. Phase II/III clinical trials with CTLA-4 and PD-1/PD-L1 immune checkpoint inhibitors are attractive therapeutic strategies in gastric cancer (GC) patients. The Cancer Genome Atlas Research network recently proposed a four-tiered molecular classification of GC, that could help to guide optimal selection of therapy. Epstein-Barr virus (EBV) positive and microsatellite unstable (MSI-high) GC are two molecular subtypes of particular interest for TIs and are frequently associated with the morphologic features of gastric cancer with lymphoid stroma (GCLS). The abundant immune infiltrate and molecular features of GCLS offer an attractive landscape to study tumour immune micro-environment, immune checkpoints and their relationship with GC cells. The aim of this study was to analyse clinico-pathological profile, EBV infection, MSI phenotype, tumour immune microenvironment and PD-L1 *status* in GCLS.

**Materials and methods:** Twenty-four GCLSs were analysed by: RNA *in situ* hybridisation for EBV (EBER), PCR/fragment analysis for MSI, immunohistochemistry (IHC) for cytokeratin (CK) AE1/AE3, CD3, CD8, double immunofluorescence (IF) for CK/PD-L1 and CD68/PD-L1 and *PD-L1* copy number alteration. PD-L1 immunoreactivity was evaluated in tumour epithelial and stromal immune cells, applying the Immunoreactivity Scoring System (IRS), as recently described. Double IF was performed in selected cases (IRS ≥7). CD3+, CD8+ T cell densities and CD8/CD3 *ratio* (CD8/CD3R) were calculated in the tumour centre (TC) and at the invasive front (IF) by digital analysis. A tissue microarray was constructed from a control group of 54 non-GCLSs and analysed by IHC for PD-L1 and by EBER.

**Results:** GCLSs included 16 EBV+/MSS, 4 EBV-/MSI-high and 4 EBV-/MSS cancers. Overall, CD8/CD3R at the IF exceeded CD8/CD3R in the TC (*p* < 0.001). CD8/CD3R was significantly higher (p = 0.008) in EBV+ than in EBV- GCLSs. PD-L1 immunoreactivity was observed at the cell membrane of tumour epithelial cells (IRS > 2) in 8/24 GCLSs and in immune stromal cells (≥1%) in 22/24 GCLSs. 3/17 cases harboured *PD-L1* amplification (IRS > 2, n = 3/3), 11/17 a normal *PD-L1 status* and 3/17 *PD-L1* hemizygous *status* (IRS > 2, n = 3/14). IRS > 2 and *PD-L1* amplification were restricted to EBV+ (6/16) and MSI-high (2/4) GCLSs. By comparison with non-GCLSs, GCLSs were more frequently EBV+ (*p* < 0.001), PD-L1+ (*p* = 0.04) and harboured a distinctive clinico-pathological profile: younger age (*p* = 0.03), upper location (*p* = 0.04), indeterminate histological subtype (Laurén classification) (*p* < 0.001), less advanced pTNM stage (*p* = 0.002) and better overall survival (*p* = 0.01).

**Conclusions:** GCLSs are characterised by distinctive clinico-pathological features, EBV infection (66.7%), high CD8/CD3R (at the IF and in EBV+ cases) and PD-L1 expression (33.3%). In keeping with the recent molecular data, PD-L1 expression (IRS > 2) and gene amplification were restricted to EBV+ and MSI-high cases, reinforcing the potential implications of immunotherapy in these molecular subtypes of GCLS.

**Support**: This work is a result of the project DOCnet (NORTE-01-0145-FEDER-000003), supported by Norte Portugal Regional Programme (NORTE 2020), under the PORTUGAL 2020 Partnership Agreement, through the European Regional Development Fund (ERDF).

### A24 Asunercept (APG101) a CD95L-neutralizing compound protects immune cells from activation-induced cell death without compromising their anti-tumour activity

#### C. Merz, J. Sykora, K. Hermann, R. Hussong, D. M. Richards, H. Fricke, O. Hill, C. Gieffers

##### Apogenix AG, 69120 Heidelberg, Germany

###### **Correspondence:** C. Merz

**Background:** Targeting immune checkpoints represents a promising immunological treatment strategy in oncology. CD95 (APO-1/Fas) is a member of the Tumour Necrosis Factor Receptor Super Family and its corresponding ligand CD95 ligand (CD95L) is frequently overexpressed in cancers and tumour-associated endothelia. Binding of CD95L to CD95 triggers activation-induced apoptosis in immune cells, making CD95 a putative immune checkpoint. Asunercept (APG101) is a fully human fusion protein consisting of the extracellular domain of CD95 and the Fc-domain of an IgG, which has been confirmed as a potent inhibitor of CD95L signalling. Here we examined the mode-of-action of Asunercept in protection of immune cells from activation-induced cell death and subsequent effects on tumour cell killing.

**Materials and methods:** Monocytes isolated from healthy-donor blood samples were differentiated *in vitro* into either M1- or M2-like macrophages. Subpopulations were analyzed for lineage-markers and cleaved-PARP by multi-colour flow cytometry following apoptosis induction by CD95L exposure. Analogous experiments were performed using PBMCs and purified T cells. Apoptosis induction in tumour cells and immune cells was also measured using a fluorogenic Caspase 3/7 substrate. Primary human microvascular endothelial cells treated with various cytokines were used as a trans-well *invitro* model of immune cell infiltration. Real-time cell analysis was performed on direct co-cultures of activated T cells and monocyte-depleted PBMCs with various tumour cell lines.

**Results:** M1- and M2-like macrophages express specific surface antigens, including CD95, following activation and differentiation of monocytes *in vitro*. Exposure of macrophages to soluble recombinant CD95L induces apoptosis in both populations, although higher sensitivity of M1- compared to M2-macrophages is observed. This is consistent with higher expression of CD95 on M1-like macrophages. Addition of APG101 protects macrophages from CD95L-induced cell death in a dose-dependent manner, but does not interfere with differentiation. Similar results were obtained after treatment of monocyte-depleted PBMCs with CD95L and APG101. Transendothelial migration of activated immune cells under immunosuppressive conditions was enhanced in the presence of APG101. Finally, in tumour killing assays using direct co-cultures of CD40-primed Ramos B cells and polyclonal T cells, tumour killing was not impaired by APG101, suggesting that primary anti-tumor killing mechanisms are CD95L-independent.

**Conclusions:** Asunercept (APG101) is a potent inhibitor of pro-apoptotic CD95/CD95L signaling in immune cells and protects activated immune cells from activation induced cell death AICD. Protection of T cells with Asunercept may enhance effector T cell migration into tumours and, importantly, anti-tumour activity induced by direct interaction between T cells and B cells is not compromised by Asunercept. The inhibition of tumour-induced CD95L-mediated apoptosis of immune cells represents an attractive and novel concept for immunotherapeutic treatment of tumours and the combination of Asunercept with co-stimulatory TNFR-SF agonists is currently being investigated.

### A25 Recognition of tumor-associated self-protein Her2 by T cells of breast cancer patients and healthy donors

#### M. P. Pinho^1^, J. A. M. Barbuto^1,2^

##### ^1^Biomedical Sciences Institute, University of Sao Paulo, Sao Paulo, Brazil; ^2^Cell and Molecular Therapy Center, NUCEL-NETCEM, University of Sao Paulo, Sao Paulo, Brazil

###### **Correspondence:** M. P. Pinho

**Background:** It is well known that T cells recognize mutated antigens in tumor cells. However, little is known about the ability of the immune system to recognize self-antigens, like Her2, a protein that is frequently overexpressed by breast cancer cells and against which therapeutic monoclonal antibodies are used in the clinic.

**Materials and methods:** We evaluated, in peripheral blood of HLA-A2^+^ breast cancer patients and healthy donors, the phenotype and number of Her2 peptide KIFGSLAFL (E75)-specific T cells. For this, circulating T cells were labeled with an E75-loaded HLA-A2 tetramer and analyzed by flow cytometry.

**Results:** Her2-specific T cells were detected in all healthy donors (490 ± 158/10^6^ cells; n = 20). Cancer patients had significantly more Her2-specific T cells (825 ± 380/10^6^ cells; n = 20; p = 0.03). No difference was observed in the median fluorescence intensity of the tetramer staining, an indirect measurement of TCR affinity. Interestingly, patients with Her2-overexpressing tumors had lower numbers of Her2-specific T cells when compared to patients with luminal B tumors that do not overexpress Her2 (Luminal B: 1532 ± 662/10^6^ cells, n = 10; Her2: 529 ± 89/10^6^ cells, n = 7; p = 0.01). When compared to healthy individuals, cancer patients had lower percentages of naïve (CD45RA^+^CCR7^+^) Her2-specific T cells (p = 0.05), but also lower numbers of naïve T cells, regardless of their specificity (p = 0.0002). Curiously, in patients, Her2-specific T cells were more frequently naïve than what was found overall in the same patient. This could be due to a tumor-specific T cell activation hindrance or to tumor-specific T cell recruitment.

**Conclusions:** Healthy and breast cancer patients have Her2-specific T cells, but with different frequency and phenotypic patterns, a phenomenon that should be further investigated to allow better results with Her2-based immunotherapy, e.g. vaccination with E75 peptides. Financial Support: FAPESP-2014/25988-1.

### A26 Effects of neoadjuvant chemotherapy on immune cell subsets in patients with breast cancer: a transcriptomic and phenotypic analysis

#### S. E. McArdle^1^, G. Foulds^1^, J. N. Vadakekolathu^1^, T. M. A. Abdel-Fatah^2^, C. Johnson^1^, S. Hood^1^, P. Moseley^2^, R. C. Rees^1^, S. Y. T. Chan^2^, A. G. Pockley^1^, S. Rutella^1^

##### ^1^Nottingham Trent University, John van Geest Cancer Research Centre, Nottingham, United Kingdom; ^2^Clinical Oncology Department, Nottingham University Hospital NHS Trust, Nottingham, United Kingdom

###### **Correspondence:** S. E. McArdle

**Background:** Breast cancer, which accounts for 13.8% of all cancer cases, remains the most common cancer and leading cause of cancer deaths in European women. Despite the improvements in patient outcome that have been achieved by earlier detection and therapeutic advances, disease continues to recur in many patients. At least in part, recurrence and therapeutic resistance could be influenced/predicted by tumour-related immunoregulatory events, and herein we examined the immunological phenotype in the periphery of patients with breast cancer.

**Materials and methods:** The profile of natural killer (NK) and immunoregulatory cell subsets in the peripheral blood of patients with breast cancer was determined by flow cytometry, as was the influence anthracycline chemotherapy on cell subset distribution in a sub-set of patients. Immune gene transcript profiles were determined in a selected subgroup of patients with luminal-A disease and triple negative breast cancer (TNBC).

**Results:** Significantly increased levels of circulating immunoregulatory T (Treg) cells and myeloid-derived suppressor cells (MDSCs) were present in the periphery of patients with cancer, and levels were positively associated with tumour burden. Treg cells from patients displayed a more suppressive/activated phenotype, as assessed by the co-expression of ICOS and CD39. The percentage of HLA-DR^neg^CD11b^+^CD33^+^ cells expressing CD15 was also greater in patients. Furthermore, levels of ‘classical’ monocytes (CD14^+^CD16^−^) were lower, but levels of ‘intermediate’ monocytes (CD14^+^CD16^++^) significantly higher in patients, with the phenotypic differences correlating with disease stage. No differences in the frequency of non-classical monocytes (CD14^+^CD16^+^) or the distribution of monocyte subsets in mono^+^ TNBC patients between patients and healthy controls were observed. The phenotypic profiles strongly correlated with the transcriptomic data. The percentage of NK cells and the proportion of the primary NK cell subsets (CD56^dim^CD16^+^ and CD56^bright^CD16^−^) in breast cancer patients and controls were not significantly different. Chemotherapy induced a significant decrease in the total B cell and increase in the monocyte count, whereas it had no effect on Treg and MDSC numbers. Chemotherapy also had no effect on NK cell counts, but induced a general increase in the expression of inhibitory and a decrease in the expression of activating receptors. Immune gene expression profiling demonstrated that three out of 12 patients with TNBC over-expressed genes related to monocyte/macrophage functions.

**Conclusions:** Although further detailed analyses in broader cohorts of patients with breast cancer are warranted, phenotypic profiling and immune gene transcript analysis of peripheral blood mononuclear cells have the potential to inform clinical decisions and help to predict therapeutic response.

### A27 Digital pathology - a key to discover new biomarkers?

#### C. Geppert, A. Hartmann

##### Institute of Pathology, Erlangen, Germany

###### **Correspondence:** C. Geppert

**Background:** The rapid growth of digital pathology in recent years opened the doors to new image analysis applications which have become an effective tool for biomarker discovery. This work wants to give an overview over the digital pathology workflow in a research application and possible approaches for image analysis. It will also introduce a novel method to increment the accuracy and robustness of the results in a reasonable time, based on pre-aligned images that help to automate a part of the analysis that is still largely manual and less objective today. This automation can lead to significant speed ups of the process and reproducible results. It can also help research organizations to deal with the growing lack of qualified pathology staff by reducing the workload of their team and help them focus on their core tasks.

**Materials and methods:** The proposed method is based on a patented image registration technology that enables the highly accurate alignment of high resolution image data. A key application of this method in biomarker discovery is the analysis of slides stained with different histochemical dyes. A first study is conducted on metastasizing melanoma in the context of personalized tumor therapy based on e.g. the PD1 antibody: The immune response against the tumor is a key indicator for the success of individualized patient treatment. To assess the immune response, specific cell groups are quantified using immunohistochemistry (IHC) markers and digital image analysis. A cohort study of >100 patients will reveal the necessary cut-offs to identify a new marker. In particular, IHC stains are created to mark T-cells, cytostatic T-cells, leukocytes, and tissue of the melanoma tumor, all on different sections. Using image registration, the sections are spatially aligned to each other and a region of interest (depicted in IHC stain for melanoma tumor tissue) is automatically transferred to all other sections.

**Results:** Following this process yields a significant speed-up, and assures a high level of interobserver agreement. Results are more objective and transparent and can be controlled completly by the digital workflow. It can serve also as a strong routine tool in research quality controll.

**Conclusions:** In conclusion, this work will give an outlook on how digital pathology contributes not only to the discovery but also to patient stratification, companion diagnostics like the immune response and to deliver personalized medicine.

### A28 Glucocorticoid receptor agonist-mediated induction of miRNA-708 modulates breast cancer proliferation and metastasis via NF-κB signaling pathway

#### K. Senthil Kumar^1^, M. Gokilavani^1^, S. Wang^1,2^

##### ^1^National Chung Hsing University, Taichung, Taiwan; ^2^Academia Sinica, Taipei, Taiwan

###### **Correspondence:** K. Senthil Kumar

**Background:** Breast cancer is one of the most common malignancies among women worldwide. This represents about 12% of all new cancer causes and 25% of all cancers in women. The microRNAs (miRNAs) are post-transcriptional regulators of gene expression and their degradation is involved in tumorigenesis. miR-708 down-regulates proliferation and metastasis of ovarian cancer cells through the glucocorticoid receptor (GR) activation and LSD1 modulation in breast cancer cells. However, the induction of miR-708 via GR in breast cancer remains largely unclear.

**Materials and methods:** Human breast cancer cell lines MCF-7 and MDA-MB-231 were subjected to investigate proliferation and metastasis under synthetic and natural GR agonistic agent dexamethasone (DEX) or antcin A (ATA), respectively. Colony formation assay, immunoblotting, immunofluorescence and real-time PCR were used to determine protein expression and localization. Luciferase reporter assay was performed to monitor the transcriptional activation of corresponding transcriptional factors.

**Results:** In this study, we found that induction of miR-708 by GR agonists DEX and ATA down-regulate proliferation and metastasis of breast cancer cell lines. Further studies suggested that GR-mediated induction of miR-708 inhabits COX-2 expression through the suppression of NF-κB transcriptional activation, which is largely involved in tumor cell proliferation. In addition, GR agonists DEX and ATA significantly inhibited tumor cell metastasis via suppression of matrixmettaloprotenases.

**Conclusions:** The present study indicate that GR-mediated induction of miR-708 may function as a tumor suppression gene in breast cancer development and miR-708/NF-κB axis may be a therapeutic interventions for breast cancer.

### A29 Hexavalent CD27 agonist HERA-CD27L enhances anti-tumour response of effector T-cells in syngeneic mouse models of colorectal cancer

#### C. Merz, D. M. Richards, J. Sykora, M. Redondo-Müller, K. Heinonen, V. Marschall, M. Thiemann, H. Fricke, C. Gieffers, O. Hill

##### Apogenix AG, 69120 Heidelberg, Germany

###### **Correspondence:** C. Merz

***Introduction:*** Tumour necrosis factor receptor superfamily (TNFR-SF) proteins are widely expressed by immune and tumour cells highlighting their importance in multiple locations and phases of the anti-tumour immune response. Agonistic stimulation of certain members of the TNFR-SF is considered to have a positive impact on immunotherapy-based concepts in clinical oncology. Apogenix has developed a proprietary technology platform for the construction of novel hexavalent TNFR-SF agonists (HERA) for the treatment of cancer. As a result of the molecular design, each molecule is capable of clustering six receptors in a spatially well-defined manner. In contrast to many agonistic anti-TNFR-SF antibodies (e.g., anti-TRAIL-R2 or anti-CD40), signaling induced by HERA-ligands *in vivo* is independent of additional crosslinking by Fc-γ receptors. The HERA engineering concept has been successfully applied to TRAIL, CD40L, LIGHT, GITRL, OX40L, 4-1BBL and CD27L resulting in hexavalent agonists suitable for further development.

***Methods & Results:*** CD27-Ligand (i.e., CD70) is a potent co-stimulatory molecule that drives T-cell activation and survival through interaction with its receptor (CD27). Here we show *in vitro* and *in vivo* data for HERA-CD27L, a hexavalent CD27 agonist. HERA-CD27L was expressed in CHO suspension cells and subjected to a lab-scale production process, including affinity chromatography and SEC-based polishing, resulting in homogenous aggregate-free protein lots. The purified protein binds its respective target-receptor with high affinity as determined by ELISA and biosensor measurements. *In vivo* stability/PK studies have been performed in addition to *in vitro* experiments with primary human and mouse lymphoid and myeloid cell populations. Specifically, HERA-CD27L was able to bind CD27 expressed on primary human CD4+ and CD8+ T-cells and importantly, binding significantly increased T-cell expansion following activation. *In vivo*, the efficacy of HERA-CD27L was evaluated in the colorectal syngeneic murine tumour models MC38-CEA and CT26. In both models, HERA-CD27L treatment resulted in a dose dependent tumour growth inhibition.

***Conclusion & Outlook:*** In summary, the data on the hexavalent HERA-CD27L demonstrate biological activity in the T-cell compartment and confers potent immune cell-driven anti-tumour efficacy *in vivo*. Our data suggest that HERA-CD27L can be applied as a single agent or in combination with immune-checkpoint inhibitors. Combinations of HERA-CD27L with other co-stimulatory ligands of Apogenix’ HERA technology platform are in preclinical evaluation. In addition, the CD95L antagonist Asunercept (APG101) represents a novel putative checkpoint inhibitor for combinatorial treatment with CD27L co-stimulation.

## Therapeutic modulation and monitoring of immune response

### A30 MuScreen^TM^ : a panel of well-characterized syngeneic models for *in vivo* screening in a high-efficient manner

#### L. Zhang, B. Mao, Y. Jin, G. Zhai, Z. Li, Z. Wang, W. Qian, X. An, M. Qiao, J. Zhang, Q. Shi

##### Crown Bioscience Inc., Taicang, China

###### **Correspondence:** L. Zhang

**Background:** Syngeneic tumor models have been revived as an effective platform, with immunotherapy becoming a research focus of oncology reagents development over the past several years. The widely used *in vitro* cell-based screens which could quickly identify responsive cells and assess PD effects, often fail in the development of immunotherapeutics, due to it targeting the complex host immune system. Alternatively, a large collection of syngeneic models with well-characterized immunotherapy panel is established to investigate PD and efficacy, but may be cost prohibitive.

**Materials and methods:** Leveraging in-house syngeneic models and its detailed profiling data, including treatment response of anti PD-1, PD-L1, CTLA-4, OX-40, GITR, LAG3 and TIM3 antibodies, RNAseq data on tumor samples, and FACS analysis on both baseline and post-treatment tumor samples, we created the first *in vivo* screening panel for immunotherapy: MuScreen. MuScreen, a 3 month screening with up to 20 well-characterized syngeneic models, including both PD and efficacy arms to be selected by the researchers based on results observed from a large dataset. Test agents from multiple clients are pooled together for each run (sharing vehicle and other common groups) providing a significant reduction in the number of animals used and the associated costs.

**Results:** CrownBio has established the first well-characterized *in vivo* panel for immunotherapy. With the three MuScreen runs, we have generated new data on common immune checkpoint inhibitors (e.g. aPD-1 antibody) and combination treatments. Base on the data of RNAseq, FACS analysis, IHC and efficacy, a bioinformatics analyses are further explored to identify the makers/pathways that could be correlated with the efficacy or PD effect of the tested IO treatment.

**Conclusions:** MuScreen, a large *in vivo* syngeneic model panel, becomes a useful tool for cancer immunotherapeutics, provides rapid screening of IO regents and may help on biomarker discovery in a cost and time efficient manner.

### A31 Proteomic test identifies sensitivity and resistance to immunotherapy in cancer patients

#### J. Weber^1^, H. Kluger^2^, R. Halaban^2^, M. Sznol^2^, H. Roder^3^, J. Roder^3^, J. Grigorieva^3^, S. Asmellash^3^, C. Oliveira^3^, K. Meyer^3^, A. Steingrimsson^3^, S. Blackmon^4^, R. Sullivan^4^

##### ^1^NYU Langone Medical Center, New York, NY, United States; ^2^Yale University School of Medicine, New Haven, CT, United States; ^3^Biodesix, Inc., Boulder, CO, United States; ^4^Massachusetts General Hospital, Boston, MA, United States

###### **Correspondence:** J. Weber

**Background:** Immune checkpoint inhibitors directed against programmed death-1 (PD-1) and cytotoxic T lymphocyte antigen-4 (CTLA-4) can provide long term benefit to a subset of cancer patients. Optimal patient selection remains challenging. Using serum mass spectrometry, we have developed a predictive test associated with prolonged survival of patients on anti-PD-1 therapy. Refinement of the test defined a subgroup of patients with particularly poor survival. Analysis of protein set enrichment analysis (PSEA) generated several hypotheses about the biological processes associated with the tests.

**Materials and Methods:** MALDI mass spectra were generated from pretreatment serum samples from 119 patients with metastatic melanoma (MM) treated with nivolumab at the Moffitt Cancer Center. A classifier development platform optimized for creation of multivariate molecular diagnostic tests which can generalize well to independent datasets was employed to create a test that identified patients with prolonged overall survival. This test classified samples into two groups: group 1 with prolonged survival and group 2 with short survival. The classifier was validated using two independent cohorts of MM patients receiving anti-PD-1 agents: 30 patients from Yale University (YU) and 25 patients, most treated with pembrolizumab, from Massachusetts General Hospital (MGH). The test was also applied to pretreatment sera collected from 21 patients from Yale that received combination PD-1/CTLA-4 blockade. The difference in outcomes between patients in group 1 and group 2 were assessed using log-rank p values and Cox proportional hazard ratios (HRs).

**Results:** Of the 119 patients used in the test set, 34 (29%) were classified as group 1. Group 1 had longer overall survival (OS) and progression-free survival (p = 0.002, p = 0.014 respectively), with two-year survival of 67%. Thirteen patients (43%) from the Yale cohort and eleven patients (44%) from the MGH cohort were classified as Group 1. In the combined analysis of validation sera sets from patients receiving anti-PD-1 agents, patients classified as Group 1 had better OS than Group 2 (p < 0.0001, HR = 0.162); their two–year survival was 86%. Within the cohort of patients receiving combination PD-1/CTLA-4 blockade, 13 (62%) were classified as Group 1. Two-year survival was 83% in Group 1 and 63% in Group 2. Further refinement of the test identified patients who had either better and worse outcome among those classified as Group 2. The difference appears to be related to proteins associated with Th1 versus Th2 mediated immunity in these patients.

**Conclusions:** A serum proteomic test identified a subgroup of patients with prolonged survival with anti-PD-1 therapy. High two-year survival in the Group 1 cohort may indicate that the test has potential utility in identifying patients who derive significant benefit from anti-PD-1 monotherapy and might gain little benefit from the addition of an anti-CTLA-4 agent. Further stratification of patients classified as Group 2 identified patients who can benefit from treatment from PD-1 monotherapy. Follow-up was 2 years for OS, and the numbers in the validation set are small, indicating that further validation is necessary. Detection of biological differences in sensitivity and resistance using PSEA methods applied to mass spectral data suggest potentially important lines of further investigation.

### A32 Number, composition and/or antileukemic activity of (DC-stimulated) invariant NKT-, NK- and CIK-cells is predictive for outcome of patients with AML, ALL and CLL

#### C. L. Boeck^1^, D. C. Amberger^1^, F. Doraneh-Gard^1^, W. Sutanto^1^, T. Guenther^1^, J. Schmohl^2^, F. Schuster^3^, H. Salih^2^, F. Babor^3^, A. Borkhardt^3^, H. Schmetzer^1^

##### ^1^University Hospital of Munich, Department of Hematopoetic Cell Transplantation, Med. Dept. 3, Munich, Germany; ^2^University Hospital of Tuebingen, Department of Hematology and Oncology, Tuebingen, Germany; ^3^University Hospital of Duesseldorf, Department of Pediatric Hematology, Oncology and Clinical Immunology, Duesseldorf, Germany

###### **Correspondence:** C. L. Boeck

**Background: iNKT/NK/CIKcell (**subsets) are important for immunesurveillance. Antibody ‘6B11’ targets the Vα24Jα18invariant-Tcell-receptor (TCR) in the CDR3-region, which is semi-invariantly rearranged in iNKTcells.

**Materials and methods:** We characterized I.) **iNKT/NK/CIKsubsets** in PB-samples from healthy donors(n = 9), AMLpts(n = 23), ALLpts(n = 20) and CLLpts(n = 21) in acute disease-stages and correlated findings with prognosis; II.) **iNKT/NK/CIKsubsets**under stimulation with Dendritic-Cells of leukemic origin (DC_leu_), generated from AMLblasts in mononuclear cells(MNC) and whole-blood(WB, containing soluble/cellular components of pts’PB) with ‘cocktails’ (DC-generating-methods/Kits).

**Results:** I.1) Compared to **healthy** MNC (significantly) ***lower*** proportions of **iNKTcells** (6B11^+^/6B11^+^CD3^+^/6B11^+^CD161^+^)**, NKcells**(CD3^−^CD56^+^/CD3^−^CD161^+^) and **CIKcells** (CD3^+^CD56^+^/CD3^+^CD161^+^) were found in MNC from AML/ALL/CLLpts. I.2) **Subtyping of iNKTcells** revealed (significantly)***higher*** proportions of CD3^+^Tcells and CD161^+^NKcells in AML/ALL/CLL expressing **6B11** compared to healthy MNC. I.3) **Prognostic evaluations** showed *higher* proportions of **iNKT/NK/CIKcells** in prognostically favorable AML-subgroups (allocation to younger age, primary disease-status, no extramedullary disease, achievement and maintenance of CR after induction-chemotherapy). Comparable correlations were seen in adultALL and CLLpts. II.1) We quantified **iNKT/NK/CIKsubsets** before/after mixed-lymphocyte-cultures (**MLC**) of T-cell-enriched immune-reactive cells stimulated with MNC/WB (with/without pretreatment ‘cocktails’ inducing blasts’ conversion to DC_leu_) from AML-pts. Our findings show, that 1)iNKT/NK/CIKcells increase after MLC independent of the stimulator-cells-suspension (under the influence of IL-2); 2)Pretreatment of MNC/WB-blasts’ with ‘cocktails’ increases iNKT-counts and induces a shift in the composition of iNKT-/NK-/CIK-subsets after MLC, that might correlate with an improved antileukemic potential; 3)INDIVIDUAL samples showed varying, however higher iNKT, CIKcellcounts after pretreatment with different (especially prostaglandin-containing) ‘cocktails’; 4)DC/iNKT/NK/CIKcells-values *after* MLC were comparable in physiological hypoxia/normoxia; 5)In cases with antileukemic blast-lytic activity after MLC T/iNKT/NK/CIKcells were significantly increased - pointing to an involvement of these cells in antileukemic reactions.

**Conclusions:** 1) Healthy MNC present with significantly higher iNKT/NK/CIKcells compared to AML/ALL/CLL-leukemic MNC. 2) Subtypes of iNKT-cells differ in healthy/leukemic samples, resembling a shift in the composition of iNKT-cells. 3) Amounts of iNKT/NK/CIKcells in AML/ALL/CLL-MNCsamples correlate with prognosis. 4) ‘Cocktail’treated AML-blasts (resulting in DC_leu_) lead to a shift in T,iNKT/NK/CIKcellcounts/compositions, what correlates with improved antileukemic activity against AMLblasts- pointing to a cross-talk of these cells. Proportions of iNKT/NK/CIKcells (based on 6B11/CD161/CD56/CD3-antibodies) should regularly be evaluated in AML/ALL/CLL-diagnosis-panels for quantitative, qualitative/prognostically relevant estimation of individual pts’ antileukemic potential in detail and to learn about their role in DC/DC_leu_triggered-immunesurveillance.

### A33 Clinical experience of nivolumab as a second line treatment of non-small cell lung cancer

#### Y. Kim, I. Oh, C. Park, S. Ahn, K. Na, S. Song, Y. Choi

##### Chonnam National University Hwasun Hospital, Jeonnam, Korea, Republic of

###### **Correspondence:** Y. Kim

**Background:** Nivolumab showed superior efficacy and survival compared to docetaxel as a second line treatment of non-small cell lung cancer. An expanded access program is ongoing in South Korea. We observed efficacy and toxicity of nivolumab and correlation with immunohistochemical(IHC) status of programmed death ligand-1(PD-L1) expression.

**Materials and methods:** We prospectively enrolled 10 patients with non-small cell lung cancer after failure of at least one line of prior systemic treatment. Cases with tissue slides, IHC stain for PD-L1 was performed with IHC 22C3 using pharmDx platform (Dako®) which is being used for pembrolizumab trial.

**Results:** Six patients with squamous cell carcinoma and 4 patients with adenocarcinoma were enrolled with male/female ratio of 5/5 and mean age of 63.8 years old. Nivolumab was used with a second, third, fourth, fifth and six line of treatment in 4, 3, 1, 1, 1 cases, respectively. IHC stain was performed in 5 cases and only 1 of them showed positive staining for PD-L1. In response evaluation after 8 weeks of treatment, there was no case showing remission, but 8 cases showed stable disease and 2 cases showed progressive disease. There was no serious adverse event except two cases with hypothyoidism after nivolumab treatment. Nivolumab was discontinued in 5 cases, and treatment is ongoing in the rest of cases. Median progression free survival from the start of nivolumab treatment is 112 days (95% confidence interval, CI: 40 ~ 183 days). Median survival was 175 days (95% CI: 131 ~ 218 days).

**Conclusions:** With these small number of cases who were heavily treated before this trial, nivolumab showed limited efficacy and poor correlation between efficacy and PD-L1 expression status. However, toxicity was minimal and further evaluation of more clinical experience is warranted.

### A34 Baseline peripheral blood biomarkers predict clinical outcome of advanced melanoma patients treated with ipilimumab: single institution real-life data experience

#### L. Fedorova^1,2^, A. Poprach^1,2^, R. Lakomy^1,2^, I. Selingerova^1,3^, R. Demlova^1,2^, K. Pilatova^1,2^, S. Kozakova^1^, D. Valik^1,2^, K. Petrakova^1,2^, R. Vyzula^1,2^, L. Zdrazilova-Dubska^1,2^

##### ^1^Masaryk Memorial Cancer Institute, Brno, Czech Republic; ^2^Faculty of Medicine Masaryk University, Brno, Czech Republic; ^3^Faculty of Science Masaryk University, Brno, Czech Republic

###### **Correspondence:** L. Zdrazilova-Dubska

**Background:** Anti-CTLA4 ipilimumab is now part of the clinical practice of advanced malignant melanoma treatment. However, only about 20% of treated patients experience a durable response, the medication is pricey and all treated patients are at risk of toxicity. The identification of patients who are most likely to experience clinical benefit is desired. Purpose of this study was to evaluate candidate baseline peripheral blood biomarkers predicting clinical outcome following ipilimumab treatment in advanced melanoma patients.

**Materials and methods:** Overall survival (OS) of a total of 51 patients with advanced malignant melanoma treated with ipilimumab at Masaryk Memorial Cancer Institute, Brno, Czech Republic was evaluated in the context of baseline routinely available peripheral blood biomarkers (LDH, CRP, relative lymphocyte count, absolute monocyte count, absolute eosinophil count). Statistical analysis was performed using survival analysis. Survival probability was estimated by Kaplan-Meier method. A difference between survival curves was assessed by log-rank test and hazard ratio (HR) was calculated, Differences at p-value < 0.05 were considered to be statistically significant.

**Results:** All patients were treated with ipilimumab after its registration approval (55% within expanded access program) in standard dosing. Median age was 61 years, 31 (61%) were female. Thirty-five (69%) individuals were stage M1c, 6 (12%) M1b, and 10 (20%) M1a. Median OS (mOS) after start of treatment was 17.3 months (95% CI 11.3; 25.9 months). The number of involved organs (0-or-1 vs. 2-and-more) at the treatment initiation was not predictive for clinical outcome. Absolute monocyte count and absolute eosinophil count were not predictive for clinical outcome. For lactate dehydrogenase, the optimal cut-off value distinguishing responders and non-responder was 5 microkat/l with mOS 19.8 months in patients with LDH <5 microkat/l and 3.5 months in patients with LDH ≥5 microkat/l (p < 0.0001, HR = 22.1, 95% CI 6.6-73.8). In low LDH subgroup, 2-year survival was 40% and 3-year survival 34%. For C-reactive protein, the optimal cut-off value was 20 mg/l with mOS 19.0 months in patients with CRP <20 mg/l and 3.2 months in patients with CRP ≥20 mg/l, (p <0.0001, HR = 12.9, 95% CI 4.3-39.0). In low CRP subgroup, 2-year survival was 36% and 3-year survival 31%. For relative lymfocyte count, the optimal cut-off value was 0.15 with mOS 19.0 months in patients with RLC ≥0.15 and 3.2 months in patients with RLC <0.15, (p <0.0001, HR = 9.6, 95% CI 3.4-27.8). In low CRP subgroup, 2-year survival was 35% and 3-year survival 31%.

**Conclusions:** Baseline routine peripheral blood parameters such as elevated LDH, elevated CRP and low relative lymfocyte count are strong predictors of overall survival in advanced malignant melanoma patients treated with ipilimumab. Stratification according to LDH level provided the strongest prediction of response to ipilimumab.

Supported by by MEYS-NPS I - LO1413.

### A35 HMGB1 plasma concentrations are associated with overall survival in lung adenocarcinoma patients

#### D. Aguilar-Cazares^1^, M. Galicia-Velasco^1^, C. Camacho-Mendoza^1^, L. Islas-Vazquez^1^, R. Chavez-Dominguez^1^, C. Gonzalez-Gonzalez^2^, H. Prado-Garcia^1^, J. S. Lopez-Gonzalez^1^

##### ^1^Instituto Nacional de Enfermedades Respiratorias Ismael Cosio Villegas, Mexico City, Mexico; ^2^Facultad de Ciencias, Universidad Nacional Autonoma de Mexico, Mexico City, Mexico

###### **Correspondence:** D. Aguilar-Cazares

**Background:** Non-small cell lung carcinoma (NSCLC) is the most frequent type of lung cancer. Within this group, lung adenocarcinoma is the most prevalent histological type. Treatment of patients diagnosed with advanced lung adenocarcinoma, whose tumors have no activating epidermal growth factor receptor (EGFR) mutations, remains in platinum-based double chemotherapy for four to six cycles. High Mobility Group Box-1 (HMGB1) is an essential activator of cellular response to genotoxicity induced by cisplatin, due to HMGB1 binds to cisplatin-DNA-adduct favoring DNA repair and chemoresistance. In contrast, some authors have reported that during cell death induced by platinum-based compounds, HMGB1 is released. In the extracellular space, HMGB1 acts as a damage-associated molecular pattern (DAMP). HMGB1 in association with other molecules released by stressed cells stimulate the anti-tumor immune response. In the present study, plasma HMGB1 concentrations from a cohort of lung adenocarcinoma patients with no activating EGFR mutations were quantified during treatment with conventional chemotherapy, and HMGB1 changes were associated with overall survival (OS).

**Materials and methods:** From 100 lung adenocarcinoma patients, plasmas were collected at the start and during distinct cycles of treatment. HMGB1 concentrations were quantified using a commercial ELISA kit. An extended Cox model was used to examine the relationship between HMGB1 plasma fluctuations and OS.

**Results:** The Cox regression analysis revealed that HMGB1 plasma concentration was significantly associated with OS in a multivariate and univariate models (p = 0.00094, hazard ratio = 0.69 and confidence interval = 0.56-0.86). The patient group that increased plasma HMGB1 concentrations showed higher OS compared to that of the other group. Results were confirmed by Kaplan-Meier survival curves.

**Conclusions:** During treatment, patients with a higher OS increased significantly plasma HMGB1 levels. The increase of plasma HMGB1, in the cohort of patient, could be due to tumor mass mainly contains malignant cells with a high sensitivity to cisplatin treatment. Our data suggest that, in NSCLC patients treated with conventional chemotherapy, the increase of HMGB1 plasma concentration could be a promising biomarker to predict a higher OS.

## Combination therapy

### A36 *In vivo* targeting of TGF-beta inhibitors to CD8^+^ T cells *via* ultra-smallamphiphilic nanoparticles

#### S. Yang^1^, K. D. Moynihan^2^, M. Noh^2^, A. Bekdemir^3^, F. Stellacci^3^, D. J. Irvine^1^

##### ^1^Koch Institute, MIT, Cambridge, MA, United States; ^2^Biological Engineering, MIT, Cambridge, MA, United States; ^3^Institute of Materials, EPFL, Lausanne, Switzerland

###### **Correspondence:** S. Yang

**Background:** Tumor specificcytotoxic T lymphocytes (CTLs) play a pivotal role in cancer immunotherapies; however, their tumor-killing efficacy is often stunted by immunosuppressive factors secreted in the tumor microenvironment. Transforming growth factor-beta (TGFβ) is one of the key immunosuppressive cytokines secreted that attenuates the effector function of CTLs. Small molecule TGFβ kinase inhibitors (TGFβi) may reverse immunosuppression and recover CTL proliferation and effector functions. However, systemic delivery of soluble TGFβi may cause severe toxicities given the pleiotropic nature of TGFβ signaling. Strategies to selectively target TGFβi to CTLs may significantly enhance therapy efficacy while reducing toxicities.

**Materials and methods:** We developeda T-cell targeted small molecule drug delivery platform based on amphiphilic gold nanoparticles (amph-NPs). Amph-NPs [1,2] are composed of 3 nm gold cores and coated by monolayers of 11-mercapto-1-undecanethiol sulfonate (MUS) and octanethiol (OT) ligands. A small molecule TGFβi, SB525334, was sequestered into the hydrophobic pockets of the alkanethiol ligand shell. To achieve *in vivo* T cell targeting, CD8 antibody fragments were chemically conjugated to amph-NPs. To assess the effect of TGFβ inhibition in CTLs on tumor progression, trivalent (gp100, trp1, trp2) cancer vaccines [3] were given to B16F10 melanoma tumor-bearing C57BL6 mice, followed by repeated dosing of systemic TGFβi, T-cell targeted TGFβi or saline control.

**Results:** We previously showed that amph-NPs penetrate cell membranes without causing transient pores or toxicity [1]. Further mechanistic studies revealed that the flexibility of the organic ligands allows amph-NPs to embed within lipid bilayers and subsequently to transit across bilayers to enter cells [2]. Here we show that we can sequester hydrophobic drugs like TGFβi in amph-NP ligand shells and promote significantly enhanced drug uptake into CD8^+^ T cells using the particles as a chaperone. By conjugating amph-NPs with targeting antibodies or antibody fragments, we transiently block their natural cell-penetrating behavior, enabling targeted uptake into specific cells. *In vivo*, antibody-conjugated amph-NPs increased CD8^+^ T cell targeting by 44-fold in spleen and 40-fold in blood 24 hours post intravenous administration. Ongoing work focuses on testing therapeutic benefits of arming CTLs with immunosuppressive-reverting drugs (TGFβi) along with a trivalent cancer vaccine that elicits robust tumor specific CTLs.

**Conclusions:** We developed a T-cell targeted small molecule immunomodulator delivery platform that has potential to reverse immunosuppression of lymphocytes caused by tumor microenvironment, and ultimately achieve enhanced anti-tumor immunity and better therapeutic outcome.

**References** [1]. Verma, A. et. al., Surface-structure-regulated cell-membrane penetration by monolayer-protected nanoparticles, Nat. Mater. 2008, 7:588–595.

[2]. Van Lehn, R. C. et. al., Effect of particle diameter and surface composition on the spontaneous fusion of monolayer-protected gold nanoparticles with lipid bilayers. Nano lett.2013, 13:4060–7.

[3]. Moynihan K.D. et al., Eradication of large established tumors in mice by combination immunotherapy that engages innate and adaptive immune responses. Nat. Med. 2016, doi:10.1038/nm.4200.

### A37 The dSLIM family of TLR9 agonists - benefit of the combination with checkpoint inhibitors in tumor models

#### B. Volz^1^, K. Kapp^1^, D. Oswald^1^, B. Wittig^2^, M. Schmidt^1^

##### ^1^Mologen AG, Berlin, Germany; ^2^Foundation Institute Molecular Biology and Bioinformatics, Freie Universitaet Berlin, Berlin, Germany

###### **Correspondence:** B. Volz

**Background:** Based on their broad activation of the innate and adaptive immune system TLR9 agonists are developed as anti-cancer therapies. The dSLIM® family of TLR agonists consists of covalently-closed dumbbell-shaped molecules acting as immune surveillance reactivators (ISR) with a broad immunomodulatory potential. One member of the dSLIM® family, lefitolimod (MGN1703), is currently under evaluation in a phase 3 trial in mCRC patients (IMPALA) and in clinical trials with other indications (e.g. patients with small cell lung cancer/IMPULSE, patients with HIV infection/TEACH). Another member, dSLIM2006, is presently in the preclinical phase of development. The checkpoint inhibitor anti-PD-1 restores T cell function especially of cytotoxic T cells and has the potential to revert immune escape of tumor cells. Since the targets of TLR9 agonists are upstream of that of checkpoint inhibitor anti-PD-1, the reactivation of the immune cells via TLR9 provides a basis for the checkpoint inhibition and thus synergistic immunological effects are expected in a combinatory approach.

**Materials and methods:** In preclinical development monotherapy with lefitolimod reduced tumor growth in several murine tumor models. For evaluation of the combinatory anti-tumor effect of lefitolimod and anti-PD-1, a syngeneic murine tumor model using subcutaneous (s.c.) inoculation of A20 cells (lymphoma) into Balb/c mice was used. In addition, the combination strategy of dSLIM2006 with anti-PD-1 was investigated in a colon carcinoma model utilizing s.c. inoculation of CT26 cells into Balb/c mice. In these models, multiple doses of lefitolimod or dSLIM2006, respectively, were given intratumorally (i.tu.) when tumor volume exceeded 100 mm^3^. Anti-PD-1 was applied by the intraperitoneal (i.p.) route.

**Results:** Injection of anti-PD-1 as monotherapy had a moderate effect on the tumor growth (tumor growth inhibition [TGI] = 20.3%) in the colon carcinoma CT26 model. While injection of dSLIM2006 led to moderate reduction of tumor growth (TGI = 32.1%) as well, treatment of mice with a combination of dSLIM2006 and anti-PD-1 resulted in a clearly increased TGI rate of 65.7% and statistically significant prolonged survival (p < 0.01). The combinatory effect of anti-PD-1 and another member of the dSLIM® family, lefitolimod, was even more pronounced: Injections of either anti-PD-1 or lefitolimod as monotherapy had a moderate anti-tumor effect (TGI = 45.9% and 49.8%, respectively) in the A20 lymphoma model. However, the combined treatment resulted in a clearly increased TGI of 99.1% and, consequently, all mice treated with the combination survived (p < 0.001).

**Conclusions:** In conclusion, we showed that the immune surveillance reactivators (ISR) lefitolimod and dSLIM2006, both members of dSLIM® family of TLR9 agonists, enhance the limited anti-tumor effects of checkpoint inhibitor anti-PD-1 in pilot studies in murine CT26 and A20 tumor models *in vivo*. These data show the promising potential for the combination of TLR9 agonists with checkpoint inhibitors.

### A38 Study of immunogenic cell death markers in lung adenocarcinoma cell lines treated with therapeutic agents

#### R. Chavez-Dominguez, D. Aguilar-Cazares, H. Prado-Garcia, L. Islas-Vazquez, J. S. Lopez-Gonzalez

##### Instituto Nacional de Enfermedades Respiratorias Ismael Cosio Villegas, Mexico City, Mexico

###### **Correspondence:** R. Chavez-Dominguez

**Background:** Lung Cancer due to high mortality is a problem of public health worldwide. Adenocarcinoma is the most frequent histotype and platinum-based double chemotherapy is employed as conventional treatment. Several studies have reported that EGFR mutation-positive patients treated with Tyrosine Kinase Inhibitors (TKI) have better clinical outcomes than these patients treated with chemotherapy. Erlotinib, a reversible TKI, induces in sensitive cell lines, cell cycle arrest and apoptosis. These biological events are also induced by Cisplatin (CDDP). Several reports indicate that some cytotoxic drugs induce a new modality of cell death called immunogenic cell death (ICD), which induces an antitumor immune response. The ICD requires a spatial-temporal expression of three essential molecules known as the hallmarks of ICD. An early event is the membrane exposition of calreticulin acting as “eat me” signal. Later, the release of cytosolic ATP to the extracellular space, and the nuclear protein HMGB1 outside the cell are required. Currently, no reports exist whether Erlotinib and CDDP induces ICD in *EGFR-*mutated and wild type lung cancer cell lines, respectively.

**Materials and methods:** Lung adenocarcinoma cell lines HCC827, HCC4006 and A549 (EGFRwild type) were obtained from the ATCC. Erlotinib was acquired from Sequoin and CDDP was obtained from Sigma. Cells cultured in 96-well plates were treated with serial dilutions of the compounds and cytostatic/cytotoxic effect was measured using MTT assay. The HCC827 and HCC4006 were treated with Erlotinib for 48 h and the A549 cell line was treated with CDDP for 24 h and the IC_50_was calculated. To study whether these cytotoxic drugs induces ICD, drug concentration that induced around 80% of inhibition was added to cell cultures. Cell death was evaluated by cell cycle analysis, necrotic and apoptotic cells were quantified by annexin-V/propidium iodide assay and fluorometry was used to evaluate whether apoptosis is caspase 3-mediated. As ICD markers, the intracellular ADP/ATP ratio (quantified by luminometry), HMGB1 (quantified by ELISA) and membrane-exposed calreticulin (detected by flow cytometry and immunofluorescence) were studied.

**Results:** In the HCC827 and HCC4006 cell lines, Erlotinib at low concentrations induced cell cycle arrest in G_0_/G_1_ phase. However, at high concentrations, apoptosis (20%) mediated by caspase 3 activation, increase of ADP/ATP ratio and of the HMGB1 release (only in HCC827) was induced. No membrane-exposition of calreticulin was detected. In A549 cells, CDDP induced apoptosis (40%) mediated by caspase 3 activation, increase of ADP/ATP ratio and of the release of HMGB1. Membrane-exposed calreticulin was detected as spots.

**Conclusions:** In *EGFR-*mutated cell lines, Erlotinib-induced cell death is not mediated by ICD. However, in the A549 cell line, CDDP induced cell death mediated by ICD.

### A39 Complete response of stage IIIB esophageal cancer combining low-dose checkpoint inhibitors with interleukin-2 (IL-2) and fever range hyperthermia

#### R. Kleef^1^, A. Bohdjalian^2^, D. McKee^3^, R. W. Moss^4^

##### ^1^Integrative Oncology, Vienna, Austria; ^2^Rudolfinerhaus, Vienna, Austria; ^3^San Diego Cancer Research Institute, San Diego, CA, United States; ^4^Cancer Decisions, Lemont, PA, United States

###### **Correspondence:** R. Kleef

**Background:** Advanced stage inoperable esophageal cancer has a poor prognosis and patients rarely enjoy durable complete response to treatment; progression free survival often is limited.

**Materials and methods:** We previously reported complete remission of far advanced lung metastasis in triple negative breast cancer at ITOC3 (Munich) 2016; here we report a similar successful treatment concept. The patient was a 56-year-old male newly diagnosed with adenocarcinoma of the esophagus with mediastinal lymphadenopathy. Histology revealed adenocarcinoma stage UICC IIIB T3 N2 with disseminated mediastinal, para-esophageal and cervical lymph node metastasis measuring up to 2.2 cm. HER-2/new score was positive. The patient refused suggested neoadjuvant CHT with FLOT. Clinically the patient presented with Karnofsky index of 90% with increasing difficulties swallowing solid food and rapid weight loss of 6 kg in the last 2 months. Therapy consisted of administration of the following combination protocol: Low-dose PD-1 immune checkpoint (IC) inhibitor nivolumab (0.5 mg/kg) with CTLA-4 IC inhibitor ipilimumab (0.3 mg/kg) administered weekly, over three weeks. This was accompanied by loco regional hyperthermia with radiofrequency fields (13.56 MHz) using the Syncrotherm device 3 times per week (max output 400 w) over the tumor region in combination with high dose vitamin C (0.5 g/kg) and alpha lipoic acid (600 mg) over three weeks. This was followed by long duration fever range whole body hyperthermia (using the Heckel device) in combination with low dose chemotherapy using cyclophosphamide 300 mg/m^2^ to down modulate T_reg_ cells. Next, moderate dose i.v. interleukin 2 (IL-2) under Taurolidine protection was administered for five days with careful titration to daily fever hyperthermia of max 39.5 °C. Herceptin in a dosage of only 4 mg/kg (patient refused higher dosage) was administered once.

**Results:** Unexpectedly, restaging 8 weeks following initiation of therapy with Gastroesophagoscopy (Upper GI Endoscopy) revealed complete response. This was confirmed by histological analysis of multiple biopsies in the former tumour bed confirming complete pathological response. At that time the patient had started gaining weight again and was free of any cancer-related symptoms. Several months later, he has regained 6 kg of weight, feels good, has no dysphagia, and continues to be monitored.

**Conclusions:** This is the second case of an advanced stage cancer patient having a complete response to primary immunotherapy treatment. Clearly, this combination immune treatment warrants further clinical studies. The authors herewith confirm that they have received consent from the patient to publish the data above.

### A40 Doxorubicin loaded immunoliposomes coupled to anti-MAGE A1 TCR-like scFv for improved drug delivery to melanoma

#### Mesha Saeed^1^, Sara Zalba^1^, Reno Debets^2^, Timo L. M. ten Hagen^1^

##### ^**1**^Laboratory of Experimental Surgical Oncology, Section Surgical Oncology, Department of Surgery, Erasmus MC, Rotterdam, The Netherlands; ^**2**^Laboratory of Tumor Immunology, Department of Medical Oncology, Erasmus MC Cancer Institute, Rotterdam, The Netherlands

**Background**

In our previous research we have exploited T cell receptor (TCR) mediated targeting through single chain variable fragments coupled to nanoparticles against melanoma. We showed that these melanoma targeted immunoliposomes show exclusive binding to antigen positive melanoma cells. Here we deploy the targeted nanoparticles encapsulating drug to target melanoma as a proof of principle. Melanoma is the deadliest form of skin cancer with increasing incidence rates. In case of metastasis, survival rates decrease from 91 to 16%. It has already been shown in previous research that drug loaded nanoparticles hold several advantages over free drugs. In our previous research, we have shown that if these nanoparticles are coupled to single chain variable fragments (scFv), against specific antigens, selective tumor uptake can be increased. For this purpose, concept of TCR-mediated targeting was exploited and scFv against MHC restricted Cancer Testis Antigens (CTA) were used that mimic TCR binding to MHC complex. The aim of this research is to formulate a targeted nanoparticle that can act as a multipurpose device, maintaining its specificity to antigens expressed by tumor cells.

**Methodology**

Anti-M1/A1 scFvs antibodies were produced, validated and coupled to nanoparticles. Coupling was optimized and post coupling validation was also performed using various methods. Model drug doxorubicin was actively loaded in liposomes prior to the coupling process. Cytotoxicity assays were performed. To prove internalization and binding of these targeted nanoparticles on melanoma cells, flow cytometry and confocal microscopy were also performed. In vivo pharmacokinetics and biodistribution was also performed in NMRI nu/nu mice. In addition to this clinical efficacy and general toxicity was also studied.

**Results:**

In vitro experiments show higher binding and internalization of immunoliposomes to antigen-positive B cells as well as M1/A1-positive melanoma cells, in comparison with non-targeted liposomes. Cytotoxicity assays showed a significant difference between targeted and nontargeted liposomal doxorubicin efficacy on M1/A1 expressing tumor cells but not on negative cells. In vivo pharmacokinetics study show, immunoliposomes are cleared a bit faster but not significantly different than non-targeted liposomes. Clinical efficacy showed slight toxicity in the targeted liposomes groups but higher anti-tumor activity as well in comparison to control groups. Biodistribution study showed higher drug levels in tumors when treated with targeted liposomes. Clinical efficacy showed a better survival and delayed tumor growth in targeted liposomal doxorubicin group.

**Conclusion:**

Targeted formulations retained 90% of the drug as well as the non-targeted formulation. Blood residence time was slightly less than non-targeted liposomes but accumulation of these targeted liposomes in tumor is high. Bioavailability of the drug can be improved by these drug loaded targeted nanoparticles. Better retention time of drug together with increased availability in tumor provides a platform for multiple purposes such as drug deliver, imaging and carrying other therapeutic molecules.

### A41 Synergistic effects of P2X7 and doxorubicin in tumor cells

#### S. Javed, J. Becher, F. Koch-Nolte, F. Haag

##### Institute of Immunology, University Medical Center Hamburg-Eppendorf, Hamburg, Germany

###### **Correspondence:** S. Javed

**Background:** The purinergic P2X7 receptor is an ATP-gated cation channel expressed by hematopoietic as well as by many tumor cells. Expression of P2X7 provides a growth advantage for tumor cells by increasing mitochondrial calcium content and stimulating cell metabolism. On the other hand, strong stimulation of P2X7 can cause death of tumor cells, and expression of P2X7 by host immune cells is detrimental to the tumor by enhancing the anti-tumor immune response. Little is known about the role of P2X7 in the context of chemotherapy. We therefore studied the effects of P2X7 stimulation on the *in vitro* toxicity of Doxorubicin (DXR) in Yac-1 lymphoma cells that express P2X7 endogenously and also in several cell lines, transduced with P2X7 variants.

**Materials and methods:** P2X7 mediated ectodomain shedding, PS exposure and pore formation were monitored by flow cytometry. Doxorubicin and ATP toxicity were observed by flow cytometry and acid phosphatase assay in lymphoma and solid tumor cell lines.

**Results:** Low doses of extracellular ATP (eATP) that caused little toxicity by themselves enhanced sensitivity to DXR by 7 to 8 fold. Transient exposure to eATP and DXR for one hour was sufficient to induce cell death after overnight culture. Mechanistically, gating of P2X7 augmented the initial uptake of DXR into cells. However, enhanced cell death was also observed even when DXR was washed away before exposure to eATP, indicating that this was not the only mechanism and suggesting that the synergism was rather due to an interaction of downstream signaling pathways. The synergism of ATP and DXR was enhanced by an agonistic nanobody to P2X7. Sensitization to DXR by eATP was also observed after transfection of P2X7 into several other tumor cell lines that do not express P2X7 endogenously. In these experiments we observed that sensitization to DXR was linked to a specific splice variant (P2X7k) of P2X7.

**Conclusions:** Our results show that P2X7 on tumor cells may contribute to the success of chemotherapy, and suggest modulation of P2X7 as a therapeutic strategy to enhance the cytotoxicity of chemotherapeutic drugs.

### A42 Cancer immunotherapy with sequential administration of trabectedin and nivolumab in advanced soft tissue sarcoma

#### E. M. Gordon^1^, K. K. Sankhala^1^, N. Stumpf^1^, W. Tseng^2^, S. P. Chawla^1^

##### ^1^Sarcoma Oncology Center/Cancer Center of Southern California, Santa Monica, CA, United States; ^2^University of Southern California Keck School of Medicine, Los Angeles, CA, United States

###### **Correspondence:** E. M. Gordon

**Background:** Trabectedin has direct cytotoxic activity in tumor cells and has also been shown to deplete pro-tumor macrophages in the tumor microenvironment (Germano et al., Cancer Cell 2013). Nivolumab inhibits the immune checkpoint molecule, PD-1, which restores anti-tumor activity in tumor-infiltrating T cells. The guiding hypothesis is that the combination of trabectedin and anti-PD-1 therapy synergistically attacks tumor cells and promotes anti-tumor immunity. The objectives are to assess the safety/toxicity and efficacy of sequential administration of trabectedin and nivolumab in patients with advanced soft tissue sarcoma (STS).

**Materials and methods:** Fourteen patients with locally-advanced and/or metastatic STS were evaluated. Each patient received 1 dose of single-agent trabectedin (1.5 mg/kg continuous intravenous infusion, CIV, for 24 hours), followed by sequential administration of trabectedin CIV and nivolumab (3 mg/kg IV over 30 minutes) every 3–4 weeks, starting 3 weeks after the first trabectedin dose. Safety/toxicity was analyzed using the NIH/NCI CTCAE v.4.03. Baseline and follow-up CT scans or MRIs were performed after every 2 cycles of the sequential chemo-/immuno-therapy. Tumor responses were assessed by RECIST v1.1 and immune-related response criteria (irRC).

**Results:** Histologic subtypes include malignant fibrous histiocytoma/undifferentiated pleomorphic sarcoma (n = 6), leiomyosarcoma (n = 3), synovial sarcoma (n = 2), myxoid liposarcoma (n = 2) and chondrosarcoma (n = 1). All patients had metastatic disease and a median of 4 lines of prior chemotherapy. Safety analysis: Grade 3 treatment emergent adverse events include anemia (n = 1), fatigue (n = 1), decreased platelet count (n = 1), and increased creatine kinase (n = 1). No immune-related adverse event has been observed so far. Efficacy analysis: Thirteen patients received 2 cycles of sequential chemo-/immuno-therapy, had follow-up CT scan or MRI, and were evaluated for objective response (OR), best overall response rate, (BORR), disease control rate (DCR), progression-free survival (PFS) and overall survival (OS). There were 2 partial responses (PR), 8 stable disease (SD) and 3 progressive disease (PD), with best overall response rate (BORR) of 15.3%, DCR of 76.9%, median PFS of >24 weeks (range: 8- > 24 weeks), median OS of >24 weeks (12.3- > 24 weeks), 6 month PFS rate of 69%, and 6 month OS rate of 92%. Six-month OS rate for all 14 patients was 86%.

**Conclusions:** Taken together, the results suggest that paired administration of trabectedin and nivolumab is safe, and that this chemo-/immuno-therapy approach has synergistic activity.

## Anti-cancer vaccines

### A43 Anti-proliferative & pro-apoptotic effects induced by simultaneous inactivation of HER1 & HER2 through endogenous polyclonal antibodies

#### N. González Suárez, G. Bergado Báez, M. Cruz Rodríguez, A. Gutierrez Pérez, L. Chao García, D. Hernández Fernández, J. Raymond Pous, B. Sánchez Ramírez

##### Center of Molecular Immunology, Havana, Cuba

###### **Correspondence:** N. González Suárez

**Background:** The epidermal growth factor receptor (HER1) and its partner HER2 are extensively characterized oncogenes and highly validated targets for cancer therapy. However, monotherapies against one of these receptors are hampering since resistance appearance, some associated with upregulation of other members of HER family. Then combined therapies using monoclonal antibodies (MAbs) and tyrosine kinase inhibitors (TKI) have been suggested as a promising strategy to circumvent resistance. We propose an alternative strategy based on simultaneous inactivation of HER1 and HER2 by multi-epitope targeting trough specific polyclonal antibodies (PAbs) induced by vaccination.

**Materials and methods:** PAbs were generated by immunizing mice with 400 μg of HER1 and HER2 extracellular domains adjuvated in a very small size proteoliposome (VSSP) derived from the outer membrane of *Neisseria meningitides*. Viability assays were performed by MTT assay, proteins expression was evaluated by flow cytometry and western blot.

**Results:** Elicited PAbs blocked receptors activation and provoked their abrupt degradation, conducing to down-signaling cascades inhibition. This effect was translated in proliferation arrest and apoptosis induction of tumor cells. Despite the differences in expression levels of HER1 and HER2 in the tested tumor lines, PAbs showed cytotoxicity in all of them, and the highest effect was obtained on cells with lower expression of both receptors.

**Conclusions:** These new insights will contribute to a rational design of HER family targeting multivalent vaccines as an encouraging approach for cancer patient’s treatment.

### A44 Development of a novel immunotherapy with Toll-like and Nod-like receptors synergy for targeted cancer therapy

#### C. Jacoberger-Foissac, H. Saliba, C. Seguin, A. Brion, B. Frisch, S. Fournel, B. Heurtault

##### Equipe de Biovectorologie, Laboratoire de Conception et Application de Molécules Bioactives, Faculté de Pharmacie, Illkirch, France

###### **Correspondence:** C. Jacoberger-Foissac

**Background:** Despite being quite effective, conventional cancer therapies have the major drawback of triggering numerous side effects. Currently, a challenging goal in this area is the development of innovative targeted antitumor immunotherapy with a long-term efficiency. In this context, our team took advantage of liposomal nanoparticle properties for the conception of cancer vaccines, which contain all the elements needed for the induction of an efficient antitumor immune response i) a peptide able to activate CD4^+^ T helper cells ii) a tumor peptide expressed by the cancer cells, recognized by CD8^+^ T cytotoxic cells and iii) Toll or Nod-Like receptor (TLR and NLR) ligand which act as adjuvants for the activation of the innate immune response. The aim of our project is to conceive liposomal vaccines, which would be effective even against tumors in later stage.

**Materials and methods:** Different vaccines formulations containing the three types of components have been designed and compared for their capacity to induce a strong immune response associated with antitumor activity *in vivo*. C57BL/6 J mice were injected in the tail vein with tumor cells (TC-1 cell line) expressing the E7 peptide, leading to the development of pulmonary tumor nodules. Then, injections of liposome-based vaccines were performed subcutaneouslyat different times after tumor implantation. One month later, lung tumor nodules are counted and the spleen and lymph nodes are harvested to perform an IFN-γ ELISpot assay against tumor cell epitopes to measure the number of tumor specific T cell.

**Results:** We evaluated three liposomal vaccines which contained the same T CD4^+^ and T CD8^+^ specific peptides but a different adjuvant. Formulations containing either ligand of TLR 2/6, ligand of TLR4 or ligand of NOD1 were able to induce a strong immune response and a good antitumor effect *in vivo* in mice grafted with pulmonary tumors. We observed an almost complete inhibition of tumor growthafter vaccine injections on days 2 and 4 after tumor implantation. Liposomes containing the NOD-1 ligand showed the most promising result: they were still effective after injections on days 6 and 8 after tumor implantation. To increase this therapeutic delay, we combined in the same nanoparticles TLR2/6 and NOD1 ligands. Surprisingly, although liposomes containing the NOD1 ligand alone induced an almost complete regression of tumors (99%) when injected at day 8 and 10 after tumor implantation, TLR2/6 and NOD1 ligand association allowed only a decrease in tumor growth of 60%.

**Conclusions:** To continue improving the efficacy of our treatment, we will test other combinations of TLR and NLR ligands in order to induce a synergistic effect. We also plan to address liposomes to immune cells by adding on nanoparticles a mannosylated ligand (which will be recognized via mannose receptor expressed by immune cells).

### A45 Phase I clinical study for validation of photochemical internalisation (fimaVacc) - a novel technology for enhancing cellular immune responses important for therapeutic effect of peptide- and protein based vaccines

#### T. Otterhaug^1^, M. Håkerud^1,2^, A. Nedberg^1,2^, V. Edwards^1,2^, P. Selbo^1,2^, A. Høgset^1^

##### ^1^PCI Biotech, Oslo, Norway; ^2^Oslo University Hospital, Oslo, Norway

###### **Correspondence:** T. Otterhaug

Background: Photochemical Internalisation (PCI) is a technology for inducing cytosolic delivery of endocytosed molecules by illumination. This is a principle (fimaVacc technology) that can be used for enhancing CTL responses; re-routing antigen presentation from MHC class II to MHC Class I by making access for the antigen to the MHC Class I presentation machinery in the cytosol of APCs. In the fimaVacc technology a photosensitising molecule (fimaporfin (TPCS2a)) is used to make endocytic membranes light sensitive, with illumination inducing permeabilisation of the membranes. The photochemical treatment also induced local inflammation that may also enhance CD4 T cell and antibody responses to vaccines.

Methods: Preclinical studies with fimaVacc has been shown to increase MHC class I antigen presentation and CTL responses in various mouse models; in addition with several antigens also helper T-cell humoral immune responses are enhanced. FimaVacc involves formulating the vaccine with fimaporfin and since peptide antigens are poorly immunogenic by themselves, an additional adjuvant is often used to induce an appropriate immune response. The fimaVACC technology has shown synergy with several adjuvants such as MPLA, poly-IC and poly-ICLC, in enhancing both cellular and humoral immune responses. A phase I clinical study with fimaVACC is ongoing in healthy volunteers in the United Kingdom. The subjects are vaccinated (up to three occasions) with models for peptide- and protein-based vaccines; HPV16 E7 peptide antigens and Keyhole Limpet Hemocyanin, using the TLR3 agonist poly-ICLC (Hiltonol) as adjuvant. The vaccine is as intradermal injections followed by illumination of the vaccination site. Local and systemic adverse effects will be assessed, and cellular and humoral immune responses will be analysed by ELISPOT and ELISA assays, respectively.

Results: The principle of the fimaVacc technology will be presented, together with preclinical results showing that the fimaVacc strongly and synergistically enhances both cellular and humoral immune responses when given with several types of adjuvants including MPLA, poly-IC, Ploy-ICLC and gm-CSF. Data showing enhancement of TNF-α and INF-γ cytokine production from both CD4- and CD8+ T cells, and the improvement of anti-tumour effects in different clinically relevant mouse models, will be presented.

Further, the design of the ongoing clinical study, and initial clinical results will be presented.

Conclusions: The fimaVacc technology strongly enhances the effect of therapeutic cancer vaccines in pre-clinical models and strong synergy is observed with several types of adjuvants. A phase I study in healthy volunteers is currently ongoing in the UK to assess the PCI technology for vaccination in humans.

PCI has a completely novel mechanism of action as a vaccination technology, representing a potent tool for stimulation of cytotoxic CD8 T-cell responses, CD4 T-cell responses as well as antibody production.

PCI can give strong synergy with commonly used immunological adjuvants.

### A46 An Immunoinformatics Framework for Identification of Personalized Tumor Epitopes in Cancer

#### T. Jaitly^1^, J. Dörrie^1^, N. Schaft^1^, S. Gross^1^, B. Schuler-Thurner^1^, S. Gupta^2^, L. Taher^3^, G. Schuler^1^, J. Vera^1^

##### ^1^Fredrich Alexander University Hospital, Erlangen, Germany; ^2^University of Rostock, Rostock, Germany; ^3^Fredrich Alexander University, Erlangen, Germany

###### **Correspondence:** T. Jaitly

**Background:** Tailor-made therapeutic vaccines is a promising approach to fight solid tumors based on individual patient’s genomics and transcriptomics profile. These personalized vaccines are actively tailored based on individual’s tumor characteristics obtained from tumor samples after surgery. A remarkable case is dendritic cell-based vaccination, an immunotherapy in which tumor epitopes targeting patient’s specific mutations are used to prime DCs, which helps in stimulating cytotoxic T cell mediated anticancer immunity once intravenously injected.

**Materials and methods:** Here we present an immunoinformatics framework for personalized identification of tumor epitopes using patient specific haplotype and mutation profiles. In the framework proposed, we analyze whole exome sequencing and RNA sequencing patient data to detect patient specific mutations and their expression level. Epitopes including the tumor mutations are computationally predicted using patient’s haplotype and filtered based on their expression level, binding affinity, tumor Variant Alelle Frequency and tumor clonality. Epitopes are further selected by calculating minimization energy for epitopes using molecular dynamics simulation.

**Results:** We predicted tumor epitopes for patients with metastatic cancer. Out of 1080 epitopes only 20 best epitopes were selected for molecular dynamics simulations to select top 10 best epitopes. These best epitopes are under experimental validation.

**Conclusions:** Our pipeline is able to predict tumor epitopes in more automatic and personalized manner.

## Adoptive cell therapy

### A47 Preclinical characterization of a PD-1-CD28 fusion receptor for T cell-based immunotherapy of Non-Hodgkin Lymphoma

#### F. Rataj, F. Kraus, S. Grassmann, M. Chaloupka, S. Lesch, C. Heise, S. Endres, S. Kobold

##### Center of Integrated Protein Science Munich (CIPS-M) and Division of Clinical Pharmacology, Department of Internal Medicine IV, Klinikum der Universität München, Munich, Germany, Member of the German Center for Lung Research, Munich, Germany

###### **Correspondence:** F. Rataj

**Background:** Interaction of the Programmed Death Receptor 1 (PD-1) and its ligand, PD-L1, has been shown to suppress T cell activity and allows tumors to evade T cell mediated immune-response. Our group recently showed that antigen-specific T cells transduced with a PD-1-CD28 fusion receptor are protected from PD-1-mediated inhibition. These cells are co-stimulated via CD28 signalling, resulting in tumor-rejections in a murine model of pancreatic cancer. The following study aims to proof the potential of PD-1-CD28 fusion receptor transduced T cells for immunotherapy of Non-Hodgkin-Lymphoma.

**Materials and methods:** Murine PD-L1 was retrovirally transduced into ovalbumine (OVA)-overexpressing murine T cell lymphoma cells (EG7). OVA-specific CD8+ were retrovirally transduced with PD-1-CD28 fusion receptor. Cytokine release, proliferation and cytotoxic activity of the transduced T cells co-cultured with EG7-PD-L1 cells were determined in vitro. Anti-tumor effect of PD-1-CD28 expressing T cells was analysed in EG7-PD-L1 tumor bearing C57Bl/6 mice.

**Results:** In vitro co-culture of PD-1-CD28 fusion receptor transduced T cells and EG7-PD-L1 tumor cells induced specific T cell activation measured by IFN-y release (p < 0.001). T cell-induced lysis of target cells was increased (three-fold induction) upon PD-L1 binding. In vivo treatment of subcutaneous EG7-PD-L1 tumor-bearing mice with transduced T cells resulted in a delayed tumor growth associated with complete tumor regressions in 40% of all mice compared to control groups. Cured mice were protected upon subsequent rechallenge with EG7-PD-L1 tumors, suggesting a memory response and epitope spreading.

**Conclusions:** Our results indicate that adoptively transferred PD-1-CD28-transduced T cells have the potential to overcome the PD-1-PD-L1 immunosuppressive axis in Non-Hodgkin-Lymphoma.

### A48 Dominant negative tumor growth factor beta receptor 2 enables T cell activity in a murine pancreatic tumor model

#### B. M. Loureiro Cadilha, K. Dorman, C. Heise, F. Rataj, S. Endres, S. Kobold

##### Division of Clinical Pharmacology, Department of Medicine IV, Klinikum der Universität München, Munich, Germany

###### **Correspondence:** B. M. Loureiro Cadilha

**Background:** Adoptive T cell transfer (ACT) with tumor-specific cytotoxic T lymphocytes is a promising treatment modality for a number of malignancies. However, so far, the success of ACT in solid tumors is poor. The lack of efficacy of ACT in solid entities is in part due to tumor-microenvironment induced T cell exhaustion and anergy. Tumor Growth Factor - β (TGF-β) is produced in large amounts by pancreatic cancer cells and their surroundings and is thought to suppress T cell activity through binding to the ubiquitously expressed TGF-β receptor-1 and −2 tetramers. We hypothesize that a truncated TGF-β-receptor may shield T cells from TGF-β effects.

**Materials and methods:** As the segment responsible for TGF-βsignaling resides in the intracellular domain of the TGF-β Receptor 2 (TGF-β-R2), a stop codon has been inserted after the tenth intracellular amino acid to prevent further translation, this receptor is also known as Dominant Negative TGF-β-R2 (DNR). The product was then cloned in the pMP71 retroviral vector. The generated receptor can be constitutively expressed in T cells upon transduction and competes with the endogenous TGF-β R2 for the formation of tetramer receptor complexes. We have expressed the DNR through retroviral transduction in primary murine T cells specific for the model antigen ovalbumin (OT-1 cells) and tested the transduced cells in vitro with cultures in the presence of TGF-β and in vivo as treatment for Panc02OVA subcutaneous tumors.

**Results:** When cultivated with TGF-β, DNR transduced cells are unaffected by TGF-β in terms of proliferation, as opposed to untransduced cells which stop proliferating in the presence of TGF-β. This is mirrored by undetectable phosphorylation of SMAD2/3 complexes in DNR T cells as opposed to untransduced T cells upon TGF-β addition. In vivo*,* ACT of DNR transduced cells in mice bearing Panc02OVA tumors, ameliorates tumor control and overall survival compared to untransduced OT1 T cells.

**Conclusions:** Adoptive T cell therapy efficacy can be improved in a pancreatic cancer model by introducing a Dominant Negative TGF-β Receptor 2 into antigen-specific T cells. This receptor may be combined with other promising strategies to improve the efficacy of cellular approaches.

